# In-Vitro Cell Culture for Efficient Assessment of Mycotoxin Exposure, Toxicity and Risk Mitigation

**DOI:** 10.3390/toxins12030146

**Published:** 2020-02-27

**Authors:** Ran Xu, Niel A. Karrow, Umesh K. Shandilya, Lv-hui Sun, Haruki Kitazawa

**Affiliations:** 1 Department of Animal Biosciences, University of Guelph, Guelph, ON N1G 2W1, Canada; rxu02@uoguelph.ca (R.X.); ushand@uoguelph.ca (U.K.S.); 2 Department of Animal Nutrition and Feed Science, College of Animal Science and Technology, Huazhong Agricultural University, Wuhan 430070, China; lvhuisun@mail.hzau.edu.cn; 3 Food and Feed Immunology Group, Laboratory of Animal Products Chemistry, Graduate School of Agricultural Science, Tohoku University, Sendai 980-8572, Japan; haruki.kitazawa.c7@tohoku.ac.jp; 4 Livestock Immunology Unit, International Education and Research Center for Food and Agricultural Immunology (CFAI), Graduate School of Agricultural Science, Tohoku University, Sendai 980-8572, Japan

**Keywords:** mycotoxins, in-vitro cell culture, toxicity assessment and mitigation

## Abstract

Mycotoxins are toxic secondary fungal metabolites that commonly contaminate crops and food by-products and thus, animal feed. Ingestion of mycotoxins can lead to mycotoxicosis in both animals and humans, and at subclinical concentrations may affect animal production and adulterate feed and animal by-products. Mycotoxicity mechanisms of action (MOA) are largely unknown, and co-contamination, which is often the case, raises the likelihood of mycotoxin interactions. Mitigation strategies for reducing the risk of mycotoxicity are diverse and may not necessarily provide protection against all mycotoxins. These factors, as well as the species-specific risk of toxicity, collectively make an assessment of exposure, toxicity, and risk mitigation very challenging and costly; thus, in-vitro cell culture models provide a useful tool for their initial assessment. Since ingestion is the most common route of mycotoxin exposure, the intestinal epithelial barrier comprised of epithelial cells (IECs) and immune cells such as macrophages, represents ground zero where mycotoxins are absorbed, biotransformed, and elicit toxicity. This article aims to review different in-vitro IEC or co-culture models that can be used for assessing mycotoxin exposure, toxicity, and risk mitigation, and their suitability and limitations for the safety assessment of animal foods and food by-products.

## 1. Introduction

Mycotoxins are toxic secondary metabolites produced by filamentous fungi that predominantly belong to species from the *Aspergillus, Fusarium,* and *Penicillium* genera. Over 500 different classes of mycotoxins have been discovered, many of which have unknown mechanisms of action (MOA) [1]. Ingestion of mycotoxins can lead to mycotoxicosis in both animals and humans, and at subclinical concentrations may affect animal production and adulterate food animal by-products. These toxic compounds of global concern are commonly detected as contaminants in a variety of commodities of plant origin, especially cereal grains, and are therefore often detected in animal feeds. Mycotoxins can also be found in animal-derived products such as meat, eggs, milk, and milk derivatives due to their carry-over from animals that have consumed contaminated feeds [2,3,4]. Natural co-occurrence of mycotoxins with potential additive, antagonistic, or synergistic effects more commonly occurs in foods and feeds than single mycotoxin contaminants [5]. Mycotoxins inflict high annual economic losses worldwide due to condemned agricultural commodities as well as reduced animal and human health [6]. Weather conditions associated with climate change have been predicted to favor more fungal contamination of foods and feeds as temperature and moisture are major factors influencing fungal growth and mycotoxin production [2,7]. Global trade of food and feed commodities contributes to the worldwide dispersal of mycotoxins [5].

A wealth of toxicity and mechanistic studies have been conducted on mycotoxins at the cellular level using kidney cells and blood lymphocytes [5,8], as well as animal performance studies [9]. However, the effects of mycotoxins on the intestine should be more thoroughly considered and assessed for the following reasons. Firstly, the intestinal epithelium is the initial site of exposure following the ingestion of mycotoxins and the first physical barrier that limits their entry into the animal [10]; damage to this barrier could also facilitate entry of luminal microbes, antigens, and other food contaminants. Secondly, the intestinal mucosa possesses the largest single compartment of the immune system underlying the epithelial lining [11]. Collectively, the gut-associated lymphoid system and intestinal epithelial cells (IECs) forming the intestinal barrier cross-talk with each other to maintain homeostasis of both the intestine and the immune system to elicit an appropriate immune response during microbial infection and to repair damaged tissues [10,12,13,14,15]. Mycotoxin exposure could render this important immunological barrier dysfunctional and this, combined with disrupted physical barrier function, could increase host susceptibility to disease. Thirdly, IECs are equipped with metabolizing enzymes and protein pumps that regulate absorption and biotransformation of xenobiotics as well as the possible efflux of metabolites back to the intestinal lumen. Mycotoxins may be able to alter the expression and activity of IEC proteins involved in absorption, efflux, and biotransformation [16], which could compromise their ability to regulate the bioavailability of other xenobiotics and nutrients. A fourth reason is that IECs may be repeatedly exposed to high concentrations of mycotoxins, which could increase the likelihood of impairment to the intestinal barrier function [17,18]. Specifically, after absorption by IECs, mycotoxins such as Ochratoxin A (OTA) and zearalenone (ZEA) could be returned to intestinal lumen either by IECs through efflux proteins, or via bile after undergoing entero-hepatic circulation [10,16,19,20]. This recirculation could result in the reabsorption of mycotoxins and prolonged exposure of IECs at the intestinal barrier, which could increase the risk of mycotoxins interacting with each other and other xenobiotics [5,21]. A fifth reason is that IECs undergo continuous renewal in order to maintain barrier function, and a number of mycotoxins are known to inhibit protein synthesis [22,23], which could impair the renewal process. A sixth reason is a potential interaction between mycotoxins and gut microbiota. Rumen and intestinal microflora are able to metabolically inactivate certain mycotoxins [9,24], however, some mycotoxins that exhibit antimicrobial activity may reduce detoxification efficiency [25,26,27]. Since the intestinal microbiota also contributes to intestinal barrier function, immune system development, and mediates the production of neurotransmitters associated with brain function [28,29,30], disrupted intestinal microbial populations could also potentially impair gut–immune and gut–brain communication.

The effects of various mycotoxins on the intestinal mucosal components have been studied both in-vivo and in-vitro. Since in-vitro cell culture models provide a cost-effective and high throughput means for the initial screening and assessment of mycotoxins, and mitigation approaches, this review will provide a summary of in vitro studies that have been carried out on individual and combined mycotoxins acting on the intestinal epithelial and gut immunological barrier using IEC and immune cell models, and explore different in-vitro IEC or co-culture models and their suitability and limitations for assessing mycotoxin exposure. The mycotoxins most commonly addressed in this review include deoxynivalenol (DON), ZEA, aflatoxin B1 (AFB1), citrinin (CIT), OTA, and mycophenolic acid (MPA) in terms of their prevalence, toxicity, and occurrence of pre- and post-harvest in animal feeds. The review also summarizes the in-vitro assessment of mycotoxin detoxifying agents (i.e. mycotoxin binders and modifiers) as feed additives that are widely used in the animal feed industry to mitigate the risk of exposure. Lastly, this review points out the need for co-culture models that are better able to more physiologically and immunologically reflect the intestinal mucosa to better assess the effects of mycotoxins on the intestine. 

## 2. Mycotoxins

Mycotoxins are structurally diverse low-molecular-weight metabolites that are chemically and thermally stable [10,31]. The three most predominant mycotoxin-producing fungi species *Aspergillus*, *Fusarium,* and *Penicillium* can be classified as either field (*Fusarium* spp. ) or storage fungi (*Aspergillus* spp. and *Penicillium* spp.) [32]; field fungi produce mycotoxins (DON and ZEA) prior to harvest [33,34], while storage fungi initially colonize plants prior to harvest and continue to grow and produce mycotoxins (AFB1, CIT, OTA, and MPA) under improper grain and silage storage conditions that favor fungal development and mycotoxin production [33,35,36,37].

DON is one of the most commonly detected mycotoxins in cereal crops such as wheat, corn, barley, and rye [38]. Various organ systems can be the targets of DON. Ingestion, for example, causes nausea and vomiting through interactions with the neural dopaminergic system; because of this, DON is also referred to as vomitoxin [39,40,41]. The immune system is another target for DON; at high concentrations, DON is an immunosuppressant, whereas, at lower concentrations, DON may stimulate the immune system [39,42]. DON also induces caspase-mediated apoptosis via activation of MAPK signaling pathways [39]. One MOA of DON includes disruption of protein translation via binding to the 60S ribosomal subunit of peptidyl transferase [43].

ZEA is another major mycotoxin produced by various *Fusarium* species. ZEA is classified as an estrogenic mycotoxin because it resembles human 17β-estradiol and binds to and activates estrogen receptors expressed mainly within the reproductive system. ZEA has been shown to also be immunotoxic, hepatotoxic, hematotoxic, and genotoxic, which may be partially attributed to ZEA contributing to oxidative DNA damage and cellular apoptosis induced by the production of reactive oxygen species [44,45,46,47,48,49]. 

Aflatoxins are produced mainly by species of *Aspergillus flavus* and *Aspergillus parasiticus* [50]. AFB1 is the most potent member of the aflatoxin family based on its well-characterized carcinogenicity leading to hepatocellular carcinoma in both humans and animals [51]. It also causes malnutrition, suppresses growth, and modulates immune function [52]. The formation of DNA adducts and the ability to cause oxidative damage might contribute to AFB1 cytotoxicity and carcinogenicity [53]. 

CIT is produced predominantly by *Penicillium* spp. [38]. Several species of *Aspergillus* and *Monascus* are also producers of CIT [36,38]. CIT naturally contaminates a variety of foods and feeds such as nuts, grains, barley, wheat, and corn, mainly during storage [6], and has been shown to be nephrotoxic in all tested animal species [38]. CIT genotoxicity is controversial, showing both positive and negative results using various in-vitro systems [54,55,56]. 

OTA is produced by *Penicillium* spp. and various *Aspergillus* spp., and it is the most prominent among the family of ochratoxins [57]. OTA has been found to contaminate cereals and cereal by-products such as wheat, rye and barley [10,38], and has been detected in animal products including pork and milk [58]. OTA is a nephrotoxin to all animal species including humans [3,59,60,61]. OTA has also been shown to be a teratogen, hepatotoxin, immunosuppressant, and carcinogen in various animal species including humans [62,63]. The number of MOAs by which OTA interferes with cellular functions has been determined. OTA inhibits protein synthesis through inhibiting phenylalanyl-tRNA synthetase, thus phenylalanine metabolism. Mitochondria are also targeted by OTA through inhibiting ATP production and inducing the production of reactive oxygen and nitrogen species. OTA also disrupts cell-cycle progression by targeting the cyclin–CDK system, disrupting mitosis, and causing chromosomal instability [64,65,66,67,68,69,70]. Lastly, OTA can also induce DNA adducts, particularly deoxyguanosine (dG) adducts [67,71,72]. 

The mycotoxin MPA is produced by *Penicillium roqueforti* fungi, which is a main silage spoiler and the most prevalent post-harvest fungi found in forage silages due to its capacity to grow in low-oxygen and high carbon dioxide as well as acidic and cold environmental conditions [37,73,74,75,76,77,78]. MPA is one of the “emerging” mycotoxins that are being detected in feedstuffs. It is frequently detected in forages, particularly silage [79]. MPA is able to inhibit B and T lymphocyte proliferation and inhibit the production of cytotoxic T cells [80,81]. MPA also exhibits antibacterial, antifungal, antitumor, and antiviral properties [80,82]. However, MPA’s immunosuppressive properties may increase the susceptibility of exposed animals to infectious diseases and sensitivity to other mycotoxins [83], and MPA’s antimicrobial properties could disrupt the normal function of the microflora in ruminants, including the detoxication of other *Penicillium* mycotoxins [84,85]

## 3. The Intestinal Barrier

### 3.1. Physical Barrier

The intestine forms a physical barrier between the microbial-rich luminal environment and sterile internal host tissues [86]. This selectively permeable barrier allows for the exchange of nutrients and antigens, while preventing the penetration of opportunistic commensal and pathogenic microorganisms and their toxins into host tissues [87,88]. The intestinal barrier consists of an external “physical” barrier mainly formed by mucous-coated IECs, and an underlying functional “immunological” barrier [13,89]. Collectively, these barriers communicate and interact with each other to maintain intestinal barrier function and optimize the outcome of the host defense against microbial infection [13,89]. 

The intestinal physical barrier is made up of a variety of polarized IECs. Five major specialized mucosal IEC lineages have been found to differentiate from multipotential stem cells located at the base of the intestinal crypts of Lieberkühn; these include enterocytes, which are the most abundant IECs [13,90], goblet cells, enteroendocrine cells, Paneth cells, and microfold (M) cells. These specialized IECs reside in different proportions at different locations along the epithelium and carry out different functions. Goblet cells contribute to intestinal physical barrier function by secreting mucus containing mucin proteins; these mucins help prevent attachment of commensal and pathogenic bacteria to the intestinal epithelium [91]. Intercellular junctions, including tight junctions (TJs) and adhesion molecules, connect adjacent IECs to maintain physical barrier function. The TJs are the major functional elements that seal the paracellular spaces; thus, they play a key role in regulating the flow of ions and small molecules and in preventing fluid leaking between the lumen and underlying tissues [92,93]. TJs are composed of transmembrane proteins and cytoplasmic scaffold proteins. The transmembrane proteins, whose extracellular domains horizontally cross the plasma membrane of adjacent cells, including occludin (OCLN), the claudin protein family (CLDNs), and the junctional adhesion molecules marvel D3, and tricellulin [92]. The cytoplasmic scaffold proteins are intracellular TJs that link the actin cytoskeleton to transmembrane TJs, and the zonula occludin proteins (ZOs) are an important group [92]. 

Commensal microbiota residing within the intestine can also contribute to the intestinal barrier function by competitively excluding attachment sites and nutrients from pathogenic microorganisms [13]. The commensal barrier is beyond the scope of this review; however, it is worth noting that commensal microorganisms can become opportunistic pathogens when the intestinal barrier function is compromised [94,95].

### 3.2. Immunological Barrier

#### 3.2.1. IECs and Intraepithelial Lymphocytes (IELs)

The intestine also possesses a functional innate and acquired immunological barrier that provides localized defense when potentially harmful luminal microorganisms, or their toxins, penetrate the host epithelial barrier. In addition to their physical barrier function discussed above, differentially specialized IECs are an important component of the host innate immune system and are considered the first line of defense provided by the immunological barrier [96]. Paneth cells, for example, are specialized secretory cells that produce large amounts of antimicrobial peptides and proteins such as β-defensins and cathelicidin, and antimicrobial enzymes such as lysozyme [96,97]. Enterocytes, in addition to their specialized role in digestion and nutrient absorption, can also serve as luminal sensors for the immune system since they possess large numbers of pattern-recognition receptors (PRRs) that are expressed both on the cell surface and within the cell. These PRRs recognize conserved structure molecules displayed on the surface of bacteria, fungi, parasites, and viruses that are referred to as pathogen-associated molecular patterns [13,15,98]. Membrane PRRs include the Toll-like receptors (TLRs), and cytoplasmic PRRs include the NOD-like receptors such as NOD1 and NOD2 [99,100]. In addition to producing mucus, goblet cells also have the capacity to take up luminal material and present antigens to dendritic cells in the lamina propria [91,101,102].

Interspersed amongst the IECs lining the epithelium are highly abundant long-living intraepithelial lymphocytes (IELs); the estimated IEL to IEC ratio in the human small intestine is 1:10 [103]. These motile cells have diverse lineages, but the majority can be broadly classified as either “unconventional” IELs that contribute to the innate immunological barrier, or “conventional” IELs that contribute to the acquired immunological barrier. Regardless of the lineage, IELs possess cytotoxic and immunoregulatory properties that are key to regulating homeostatic crosstalk between the innate and acquired immunological barriers and commensal microbiota, and their dysfunction has been implicated in gastrointestinal disease [104].

#### 3.2.2. Lamina Propria

The subepithelial lamina propria is the site were effector immune cells of the intestinal immune system can be found [105], these include numerous innate immune cells such as macrophages (M_∅_) and innate lymphoid cells (ILCs) and the acquired immune lymphoid B and T cells [105]. The intestine is known to represent the largest pool of tissue M_∅_ in the body [106,107]. These M_∅_ are innate immune effectors with potent phagocytic and bactericidal activities [11,96,108], but unlike other macrophage populations, they inflict minimal inflammatory collateral tissue damage [13,96]. This distinct property could be ascribed to high expression of phagocytosis-promoting genes such as Mertk, Cd206, Gas6, Axl, Cd36, Itgav, and Itgb5 [109,110], as well as low or lack of expression of receptors associated with innate immune activation, such as receptors for LPS (CD14), Fcα (CD89), Fcγ (CD64, CD32, and CD16), CR3 (CD11b/CD18), and CR4 (CD11c/CD18) [108]. This special functional adaption of resident intestinal M_∅_ allows them to maintain local tissue homeostasis in part by preventing excessive immune reactions to commensal microbiota and food antigens that might otherwise elicit chronic inflammation and tissue damage [111]. Resident intestinal M_∅_ also efficiently remove apoptotic cells and foreign debris and contribute to the repair and remodeling of damaged tissues [112,113]. Collectively, these unique properties ascribe intestinal M_∅_ to the maintenance of intestinal homeostasis [112,113]. 

In addition to M_∅_, a large population of ILCs is also located within lamina propria [114]. This mixed population of ILCs can be classified based on their cytokines and transcription factors, including natural killer cells, ILC1, ILC2, or ILC3 [115,116,117,118]; respectively, these ILCs are phenotypically and functionally similar to the T helper (T_H_) cell subpopulations T_H_1, T_H_2, and T_H_17 [114]. These ILCs have cytotoxic and immunoregulatory properties that allow them to rapidly respond to and orchestrate the host response against gastrointestinal threats and also maintain epithelial integrity and tissue homeostasis [119]. 

A number of acquired lymphoid cell populations are also found within the subepithelial lamina propria. Both conventional CD4^+^ and CD8^+^ T cells are found and they perform immunoregulatory and cytotoxic activities, respectively [105,120,121]. Immunoglobulin (IgA)-producing B1 cells are also present [105]; the IgA antibodies produced by their differentiated plasma cells are selectively transported across the epithelium into the intestinal lumen where it helps to prevent microbial invasion by decreasing their motility and adhesion to the surface of the epithelium [122,123].

#### 3.2.3. Gut-Associated Lymphoid Tissue (GALT) and Mesenteric Lymph Nodes (MLNs)

A series of events must occur in order for the above-mentioned acquired lymphoid cells to become activated and drive an acquired immune response. M cells must sample and transfer luminal antigens and intact microorganisms to underlying M_∅_ and dendritic cells, which then migrate the Peyer’s patches (PP) of the gut-associated lymphoid tissues (GALT) and/or draining mesenteric lymph nodes (MLN) where they convene with T_H_ cells to carry out antigen presentation. Although the events are less well characterized in the PP than MLN, it is believed they are analogous [124]; in short, antigen-specific T_H_ cells become activated during antigen presentation, and then can assist with the activation of antigen-specific B cells, which clonally expand within germinal centers and differentiate into IgA secreting plasma cells.

### 3.3. Cross-Talk between IECs and Immune Cells

Bidirectional communication between IECs and immune cells facilitates protection from microbial invasion, tissue homeostasis, and repair. This cross-talk is mediated in part by secreted cytokines [12,13,14,15], many of which are commonly produced by immune cells and IECs [10,14]. Some of these cytokines include TGF-α, IL-1, IL-10, IL-15, IL-8, IL-1α, and β, IL-6, TNF-α, MCP-1, CCL20, and GM-CSF [14,125]. IECs also possess receptors for various cytokines, which allows them to respond to immune cell signals. For example, it has been reported that goblet cell mucus production and properties can be directly affected by IL-10 produced by M_∅_ and T-cells within the lamina propria [126]. The expression of IEC TJ proteins can also be inhibited by TNF-α, IFN-γ, and the interleukins IL-2, IL-4, and IL-8, which results in increased gut paracellular permeability [127]. Also, the binding of IL-1 to its receptor on IECs amplifies the secretion of IEC pro-inflammatory cytokines [128].

## 4. In-vitro Intestinal Epithelial Barrier Models

Although primary cells are most biologically and physiologically similar to the gastrointestinal epithelial barrier, their short-life span and rapid loss of differentiated characteristics limit their use for in vitro studies [129,130]. Instead, immortalized cell lines of animal and human origin including Caco-2, IPEC-1, and IPEC-J2 IECs have been extensively utilized as in vitro models of the intestinal epithelium to study the effects of mycotoxins on intestinal barrier function. These cell lines have been used at various differentiation states, proliferative versus differentiated, for example, to simulate different intestinal microenvironments [131,132]. Undifferentiated IECs present as a tumorigenic phenotype and do not display cell polarity [131,133]; they also appear similar to dividing cells in tissue undergoing regeneration or repair after damage [132,134]. In contrast, differentiated cells mimic the mature small intestinal barrier in that they have defined epithelial characteristics such as TJs and microvilli, which are lacking in undifferentiated cells [135]. 

### 4.1. Caco-2

Human Caco-2 cells have been the most widely used IEC line in recent decades [136]; Caco-2 is a cancer-derived cell line originally isolated from a human colon adenocarcinoma [137]. However, once differentiated after 18–21 days of culture post-confluence, they become a homogenously polarized monolayer of enterocyte-like cells with apical and basolateral membranes, a brush border with microvilli and TJs [138,139,140,141]. Caco-2 cells have also been shown to express TLRs and produce various cytokines [140,142].

### 4.2. IPEC-1 and IPEC-J2

IPEC-1 and IPEC-J2 are two other IEC lines that have been used as in vitro models of the intestinal barrier. Both IPEC lines were derived from the porcine small intestine. IPECs are spontaneously immortalized non-transformed and non-carcinoma cell lines, established from normal IECs [143,144]. IPEC-1 was derived from the jejunum and ileum of piglets less than 12 hours old. The IPEC-J2 cell line was originally isolated from the jejunum of neonatal unsuckled pigs [139,143,145]. Both IPECs are able to spontaneously differentiate into multiple IEC types [139], and a continuous polarized monolayer and TJ structure can be formed after differentiation [144]. Compared to Caco-2, the IPEC-1 and IPEC-J2 IECs attain a homogenous appearance and express various differentiation markers within a shorter period of time; within 10 days and 1–2 weeks of culturing post-confluence, respectively [143,146], and the IPEC-J2 line is more morphologically and functionally differentiated than the IPEC-1 line [144]. Since the pig intestine closely resembles the human intestine genetically and physiologically, IPECs have been used to model the human intestinal barrier [129,144].

### 4.3. In-vitro Cell Culture Systems

In vitro models of the intestinal epithelial barrier have traditionally consisted of one-dimensional (1D) monolayers grown on the surface of culture vessels. With this 1D culture system, functional IEC characteristics, such as cell polarity, are not well defined [147], and misleading results can possibly be obtained. Alternatively, a two-dimensional (2D) monoculture system can be achieved by growing IECs on microporous permeable membrane supports [148,149]; this system structurally mimics the apical and basolateral sides of the intestine and leads to the development of polarized IECs. Most reviewed cytotoxicity studies (Table 1) have been conducted using a 1D monoculture system and the 2D monoculture system has been extensively applied to study the effects of mycotoxins on intestinal barrier function parameters such as transepithelial electrical resistance (TEER) and TJ protein expression. 

More complex 1D and 2D co-culture systems that are more physically and functionally similar to the intestinal barrier have also been established. These co-culture systems involve cultivating more than one cell type together within one culture system [129,150,151]. The IEC + immune cell co-culture system involving permeable membrane supports is one such established 2D co-culture model [140]. 

## 5. Effects of Selected Mycotoxins on Intestinal Barrier Function

### 5.1. Cytotoxic Effects of Individual or Combined Mycotoxins on IECs

#### 5.1.1. Individual Mycotoxins

The cytotoxicity of selected mycotoxins has been evaluated using various IEC models on the basis of cell viability (Table 1) as well as proliferation at different concentrations and exposure durations. Mycotoxin cytotoxicity is usually the first parameter to be measured, not only to evaluate the cytotoxicity, but also to identify appropriate mycotoxin concentrations that can be used in follow-up experiments of intestinal barrier function. 

The cytotoxicity of DON on different IEC models has been the most studied among the five reviewed mycotoxins. Results using various IEC models including Caco-2, IPEC-1, and IPEC-J2 have shown that DON induces cell death at various concentrations and under different durations of exposure (Table 1). A wide range of DON exposure concentrations have been used, ranging from as low as 0.0001 up to 100 μM [156].

A variety of cytotoxicity assays have been applied to assess the cell viability (Table 1) and tetrazolium salt MTT (3-[4,5-dimethylthiazol-2-yl]-2,5-diphenyltetrazolium bromide) was most widely used in the reviewed studies, which was shown to be a quick and suitable assay to detect a wide range of mycotoxins for different cell types [171]. In order to avoid false results, more than one cytotoxicity assay has been applied in parallel in some studies, and both similar and discrepant results have been reported [143,154,164]. The 3-(4,5-dimethylthiazol-2-yl)-5-(3-carboxymethoxyphenyl)-2-(4-sulfophenyl)-2H-tetrazolium, inner salt (MTS), and Neutral Red (NR) assays have been reported to yield similar cell viability results based on Caco-2 cell viability after 24 h DON exposure [154]. The authors in [164] observed similar cell viability results from the 2,3-Bis-(2-Methoxy-4-Nitro-5-Sulfophenyl)-2HTetrazolium-5-Carboxanilide (XTT) and NR assays in IPEC-1 cells. However, [143] reported that the lactate dehydrogenase (LDH) leakage assay was less sensitive to DON toxicity compared to the NR assay using both IPEC cell lines. Interestingly, the route of application, apical versus the basolateral side of IECs, appears to influence DON-mediate changes in cell viability, as IPEC-J2 cell viability was more significantly affected when DON is applied to the basolateral side [157]. This differential susceptibility of apical and basolateral surfaces of IECs to DON exposure could be attributed to their biological and functional distinctions such as different protein and lipid compositions [172]. It could also result from the addition of the mucus produced by IPEC- J2 cells covering the apical side of epithelial monolayer [139] as an extra defense line against DON exposure. 

The differentiation status of cells may also influence sensitivity to DON exposure. It has been reported that dividing IPEC-J2 and Caco-2 cells, for example, are both more susceptible to DON than their differentiated counterparts [132,154,159]. Differentiation status is typically defined by cell culture duration in some of the reviewed cytotoxicity studies. For example, Caco-2 cells and IPEC-J2 grown on microplates without membrane inserts less than 4 days after seeding were considered undifferentiated, whereas, cells cultured at least 17 days post-seeding were considered differentiated [153,154]. 

Cell death has been reported to be induced by DON via both apoptosis and necrosis. Caspase 3, a marker for induction of apoptosis, was activated only at a high concentration of DON (6.7 μM) in both IPEC-1 and IPEC-J2 cell lines indicating DON-induced apoptosis [143]. However, necrosis-induced cell death was observed in both differentiated and undifferentiated IPEC-J2 cells after 72 h exposure to DON (0–67 μM) and it was found that the proportion of necrotic cells was concentration-dependent [158,159]. 

ZEA has also been reported to have adverse effects on IPEC-1, IPEC-J2, and Caco-2 cell viability at various concentrations after different exposure durations (Table 1). ZEA is less toxic than DON based on viability studies performed by [156,161]. ZEA appears to induce apoptosis via mitochondrial damage by reducing antioxidant enzyme activities; this may lead to an accumulation of ROS and decreased mitochondrial membrane potential [46]. Necrosis-induced cell death in undifferentiated and differentiated IPEC-J2 cells was also observed after 72 h of exposure to ZEA at 19.9–99.5 μM [158]. 

AFB1 induced a concentration-dependent decrease in the viability of both undifferentiated and differentiated Caco-2 cells between 24 h and 72 h of exposure [165]. In contrast to DON, the differentiated Caco-2 cells were found to be more susceptible to AFB1 than undifferentiated cells after 72 h of exposure, which could be due to more metabolic and transport enzymes being expressed by the differentiated mature enterocytes [165]. A similar concentration-dependent decrease in Caco-2 cell viability was also observed by [166] after 24 h exposure to a range of AFB1 concentrations (0–100 μM) with LC50s reported to be 5.39 and 6.02 μM obtained from MTT and LDH assays, respectively. AFB1 LC50s obtained from NR assay have also been reported by [167] after Caco-2 cells were exposed to AFB1 (0–100 μM) for 24, 48, and 72 h, which were 10, 2, and 0.5 μM, respectively.

Limited results have been reported on the effects of CIT, MPA, and OTA on cell viability compared to DON and ZEA. CIT has been reported to reduce human HCT116 colon cancer cell viability after 36 h exposure at concentrations of 150 and 300 μM, with the identified LC50 being 300 μM [169]. Authors in [168] reported that CIT exposure also resulted in a decrease in Caco-2 cell viability at 399.6 and 999 μM after 48 h exposure. Cell apoptosis was induced by CIT via endoplasmic reticulum stress [169]. Concerning MPA, [75] reported a concentration-dependent cytotoxic effect of MPA after 48 h exposure in both undifferentiated and differentiated Caco-2 cells, however, LC50 was not obtained with the tested concentration range. However, based on the LC20 calculated in the study, undifferentiated Caco-2 cells appeared to be more susceptible to MPA than differentiated cells [75]. Lastly, the cytotoxic effect of OTA was concentration-dependent, and two LC50s have been reported for the Caco-2 cell line after 24 h of OTA exposure using MTT and LDH assays; these were 21.25 and 16.85 μM, respectively [166]. However, a significantly different OTA LC50 of 145.36 μM was recently reported after 24 h of exposure using Caco-2 cells [170]. 

Mycotoxins have also been reported to affect cell cycle progression and proliferation. The proliferation of IPEC-1 and IPEC- 2 cells have been reported to be stimulated at lower DON concentrations or inhibited at higher concentrations [143]. For example, 0.67 μM of DON stimulated IPEC-1 cell proliferation after 48 h exposure and stimulated the proliferation of both IPEC-1 and IPEC-J2 cells after 72 h of exposure [143]. However, it is inconclusive whether the stimulated proliferation of IPEC cells resulted from a primary effect of DON or a secondary effect from DON-induced cell death [157] as almost all types of epithelial cells forming monolayers are capable of undergoing self-repair after injury by inducing cell proliferation and migration to the injured site [173].

DON started to inhibit the proliferation of IPEC-1 cells at higher concentrations ranging from 3.4 to 6.7 μM after 48 and 72 h of exposure [143]. The same pattern of effect was also observed for IPEC-J2 cells after 72 h of exposure [143]. The route of application of DON, the apical versus the basolateral side of IECs for example, has also been reported to influence IPEC-J2 cell proliferation. The authors in [157] reported IPEC-J2 proliferation was more significantly stimulated when DON is applied to the basolateral side. Selected mycotoxins other than DON, only OTA has been investigated and it inhibited the proliferation of Caco-2–14 and HT-29-D4 cells by 50% (IC50) at 30 and 20 μM, respectively [174].

At a higher concentration of 6.7 μM, DON decreased the percentage of IPEC-J2 cells in the G0/G1 phase after 24 [160], 48, and 72 h of exposure [143,157]. The authors in [160] also observed a decrease in the percentage of IPEC-J2 cells in the G0/G1 phase at a lower DON concentration of 0.67 μM after 6, 12, and 24 h of exposure. A prolonged IPEC-J2 cell G2/M phase was also induced by DON after 12 and 24 h [160] and 48 h of exposure at 6.7 μM [143]. However, a decrease in cell percentage in the G2/M phase was observed by [160] after 12 h exposure of DON at a lower concentration of 0.67 μM. A prolonged S phase in IPEC-J2 cells was also reported after 6, 12, and 24 h exposure to DON at 0.67 μM, but S phase was reduced at 6.7 μM after 12 h exposure [160]. 

DON also induced a reduction in the percentage of cells in the G0/G1 phase and G2/M arrest in IPEC-1 cells after 48 and 72 h of exposure at 6.7 μM [143]. A prolonged S phase was also observed in IPEC-1 cells by [143] after 48 h exposure of DON at 6.7 μM. The results on cell proliferation and cell cycle distribution should be considered integrated with the interpretation of the effect of DON on cell growth. The cell cycle shift from G0/G1 to S and G2/M phases and increase in cell proliferation [173] induced by DON at lower concentration might indicate intestinal epithelial cells were undergoing self-repair after DON-induced injury; whereas exposure to higher concentration of DON could have negative impact on intestinal epithelial cell growth by inducing G2/M arrest [175] that allows the cell to repair the DNA damage or misaligned chromosomes at the mitotic spindle [176]. 

#### 5.1.2. Mycotoxin Combinations

Mycotoxin mixtures have also been explored for their effects on IEC cytotoxicity. Concerning DON + ZEA, one of the most prevalent mycotoxin combinations, [161] observed antagonism of DON + ZEA mixtures on IPEC-J2 cell viability after 48 h exposure at both tested exposure combinations (2 μM DON + 40 μM ZEA and 0.5 μM DON + 10 μM ZEA). The authors in [177] also observed an antagonistic effect of DON + ZEA (100 μM/ 40 μM) on HTC116 human cell viability after 24 h exposure, whereas [155], reported that all three combinations of DON + ZEA (10/10, 10/20, and 20/10 μM) resulted in a significant reduction in Caco-2 cell viability compared to individual mycotoxins. With the combination of 10 μM DON + 10 μM ZEA, [164] also observed that the mixtures of ZEA and DON elicited synergistic effects on Caco-2 cell lipid peroxidation and antagonistic effects on DNA synthesis. Lastly, the viability of THP-1 immune cells in a Caco-2 + THP-1 co-culture model was decreased after 48 h exposure to DON + ZEA mixture (LC30/LC30, which was not specified in the article) [151]. 

### 5.2. Mycotoxins and Intestinal Permeability

Intestinal permeability is one of the key features reflecting the ability of the intestine to function as the barrier [178]. TEER is commonly used to assess IEC permeability in vitro, and a reduction in TEER has been used as an indicator of mycotoxin-induced epithelial damage [152]. Non-cytotoxic concentrations of tested mycotoxins were usually chosen for TEER studies in the reviewed studies to eliminate the effect of uncontrolled cell death on a reduction in TEER [179].

Paracellular tracer flux assays are often applied following the measurement of TEER to investigate if the potential cause of the observed decrease in TEER is increased intestinal epithelial paracellular permeability. The most commonly applied in vitro paracellular markers include fluorescence compounds (e.g., lucifer yellow, LY), or fluorescent-labeled compounds such as fluorescein isothiocyanate (FITC)-dextran and FITC-insulin [180]. As TJs are the major functional components to regulate the paracellular pathway [181], the assessment of TJs at both gene and protein levels can also be performed to further investigate the mechanism by which compromised intestinal barrier function is induced by mycotoxins. 

#### 5.2.1. Measurement of Transepithelial Electrical Resistance (TEER)

The impact of DON on TEER has been extensively studied using various in vitro intestinal models. DON decreases TEER values in both concentration- and time-dependent manners regardless of in vitro models used [152,157,182]. The lowest DON concentration that reduced Caco-2 cell TEER values was 0.17 μM after 24 h exposure [183]. A decrease in Caco-2 TEER measurements has also been observed in a Caco-2+ THP-1 co-culture model after 48 h exposure to DON at LC10 and LC30 [151]. In addition to concentration and exposure duration, DON-mediated changes in TEER also depend on cell type. It has been reported that DON reduced IPEC-1 cell TEER measurements more significantly than Caco-2 cells, indicating that IPEC-1 cells are more susceptible to DON exposure [184]. The route of DON application can also affect TEER readings, as it has been reported that the decrease in Caco-2 and IPEC-J2 cell TEER measurements was more pronounced when DON was applied to the basolateral side compared to the apical exposure of DON [143,152]. The authors in [151] reported a decrease in Caco-2 cell TEER readings in a Caco-2 + THP-1 co-culture model after 48 h exposure to both ZEA at LC10 and LC30 (LC10 and LC30 not specified in the article). In a monoculture system, [164] observed that ZEA reduced TEER readings at a concentration of 50 μM over 10 days of exposure duration.

The impact of other mycotoxins on IEC TEER measurements has been less well studied than DON. A decrease in TEER was induced by OTA in Caco-2 and HT-29-D2 cell models [166,174,185,186]. The route of application of OTA can affect TEER readings, as it has been reported that the decrease in HT-29-D2 TEER measurements was more significant and rapid when OTA was applied to the basolateral side compared to the apical exposure of OTA [174]. However, [185] observed the equal toxic effect of OTA on Caco-2 cell TEER measurement on both apical and basolateral exposure. The authors in [186] reported that TEER decrease in Cacao-2/TC7, a clonal derivative of parental Caco-2 cells induced by 48 h exposure to OTA at a concentration up to 200 μM was reversible and the TEER value was fully recovered within 24 h after mycotoxin exposure cessation. AFB1 at 100 μM also decreased the TEER values in Caco-2 cells after 7 days of exposure [166]. Lastly, MPA has also been reported to induce decreased TEER in the Caco-2 cell model after 21 days of continuous exposure at the highest concentration of 190 μM [75]. 

There are limited data on the effects of mycotoxin mixtures on IEC TEER measurements. Caco-2 cell TEER measurements in the co-culture model were reported to decrease after 48 h exposure to DON + ZEA mixture of two different ratios (LC10/LC10 and LC30/LC30), respectively (LC10 and LC30 were not specified in the article) [151]. 

Although TEER values that have been reported in the literature have been corrected for the surface area of the membrane inserts used and is typically reported in units of Ω *cm^2^ [187], other factors that may have impact on TEER measurements should also be considered for purpose of interlaboratory comparisons, including temperature, cell passage number, the composition of cell culture medium, and duration of cell culture [188]. 

#### 5.2.2. Assessment of the Expression of TJ Proteins

DON exposure has been reported to induce alterations in the expression of TJs at both gene and protein levels in various in vitro IEC models and contradictory effects have been reported (Table 2). Up-regulation of gene expression has been often observed, whereas a decrease in protein expression has been reported (Table 2). The reduction in protein levels associated with the rise in mRNA levels could indicate a compensatory mechanism in place for repair [183,189]. The inconsistent findings emphasize that analyses of mRNA and protein expression should be performed in parallel since the mRNA level does not necessarily predict the amount of protein [190,191]. 

In contrast to DON exposure, [166] observed a decrease in mRNA expression of CLDN3 and OCLN in Caco-2 cells after AFB1 exposure (0–30 μM), but no changes in CLDN4 mRNA expression was observed. OTA exposure decreased in Caco-2 cell mRNA expression of CLDN3, CLDN4, and OCLN [166]. This inhibitory effect at the transcription level could be explained by its ability to form DNA adducts [67,71,72]. A decrease in protein expression of CLDN3 and CLDN4 has also been reported [185,192]. 

#### 5.2.3. Measurement of Flux of Paracellular Markers

DON induced a dose-dependent increase in the apical to basolateral transport of fluorescence compounds LY and 4 kDa FITC-dextran in Caco-2, IPEC-1 and IPEC-J2 cell lines [152,189,193]. The results on the paracellular passage of 4 kDa FITC–dextran also indicated that IPEC-1 exhibited more sensitivity to DON than Caco-2 cells [184]. 

It has also been reported that OTA did not affect IEC permeability to 20 and 40 kDa FITC-dextran [194], indicating that the intestinal epithelial cells still partially retain their barrier function during OTA exposure, and that larger molecules are selectively excluded, which is also in agreement with DON exposure.

### 5.3. Effects of Mycotoxins on Translocation of Intestinal Microorganisms

In addition to increased intestinal permeability and dysfunctional mucosal immune system, impaired intestinal barrier function is also associated with translocation of luminal antigens [92,178,195,196], which is another endpoint that has been used to investigate the effects of mycotoxins on the intestinal barrier. In vitro studies have shown that DON promoted transepithelial passage and invasion of *Salmonella typhimurium* in both differentiated and undifferentiated IPEC-J2 cells; the increased transepithelial passage of *S. typhimurium* was concentration-dependent [132]. DON also increased the transepithelial passage of *Escherichia coli* in IPEC-1 and IPEC-J2 cells [184,189]. MPA did not promote non-invasive *E. coli* to cross the intestinal epithelium in an in vitro study with Caco-2 cells [75]. 

## 6. Effect of Selected Mycotoxins on the Intestinal Immune System

Selected mycotoxins also have cytotoxic effects on immune cells. A 0.85 μM DON induced apoptosis in the RAW264.7 macrophage cell line [197]. The apoptosis of Jurkat human T cell line was induced by DON in the concentration range tested (0.85–3.4 μM) [198]. Concerning *Penicillium* mycotoxins, CIT, OTA, and MPA induced cell death and inhibited proliferation of bovine macrophage cell line (BoMacs) in a concentration-dependent manner [199]. A decrease in cell viability of THP-1 cells, human leukemia monocytic cell line, was observed in a Caco-2+THP-1 co-culture system after 48 h exposure to ZEA LC30 (LC30 not specified in the article) [151]. 

Host mucosal immune response to the invasion of luminal antigens/pathogens requires coordination between IECs and immune cells. Being part of the intestinal innate immune system, IECs serve as dynamic sensors for luminal microbes by expressing PRRs such as TLRs. IECs can also direct the mucosal immune response by producing important chemokines and cytokines that are responsible for the recruitment of immune cells and the induction of the inflammatory response [10,93].

Measuring the expression of cytokine and PRR at gene or protein level has been an endpoint that is commonly used to evaluate the effects of selected mycotoxins on the intestinal immune system using in vitro IEC models. At 2 μM of DON exposure, DON has been reported to up-regulate the expression of IPEC-J2 cell IL1-α, IL1-β, IL-6, IL-8, TNF-α, and MCP1 genes after 48 h of exposure, whereas, 0.5 μM exposure simulated expression of IL1-β, IL-6, and IL-8 genes, and down-regulated expression of IL1-α and MCP1 genes [200]. The up-regulatory effect of DON on IL-6, TNF-α, and IL1-β through the NF-κB pathway was also observed in IPEC-J2 cells after 24 h of DON exposure within the concentration range of 0.34 μM to 6.7 μM [162]. DON also reportedly induced a concentration-dependent increase in the secretion of IL-8 protein by Caco-2 cells through NF-κB after 48 h exposure [201], and [194] reported an increase in IL-8 protein secretion by Caco-2 cells after 12 h, which was associated with NF-κB, PKR, and p38 pathways.

The effect of ZEA on the modulation of cytokine gene expression was carried out using the IPEC-1 and IPEC-J2 cell lines. At a higher concentration of 40 μM, ZEA up-regulated IL1-α, IL1-β, IL-6, IL-8, TNF-α, and MCP1 after 48 h exposure by IPEC-J2 cells, whereas ZEA at 10 μM only stimulated the gene expression of IL1-α, IL1-β, and IL-8 [200]. A stimulatory effect on IFN-λ and IL-4 gene expression was observed in IPEC-1 after 1 h of exposure to 25 μM of ZEA [23]. However, contradictory results have also been reported in other studies. The authors in [163] observed no significant effects on the expression of assessed cytokine genes after IPEC-1 cells were exposed to 10 μM of ZEA for 24 h, including TNF-α, IL1-β, IL-6, IL-8, IL-12p40, IFN-λ, MCP1, IL-10, IL-18, and CCL20, and 10 μM and 25 μM of ZEA exhibited no effect on the expression of IPEC-1 cell IL-8 and IL-10 genes after 24 h of exposure [164].

As for the effect of other mycotoxins, in Caco-2 cells, the protein expression of IL-8 was stimulated in a concentration-dependent manner by MPA at concentrations ranging from 78 μM to 780 μM after 48 h of exposure [75]. Whereas, [194] reported OTA did not have a significant impact on protein secretion of IL-8 in Caco-2 cells. Lastly, [23,163] observed an increase in the expression of TLR2, TLR3, TLR4, and TLR8 genes in IPEC-1 cells after 10 h exposure to 10 μM of ZEA and 1 h exposure to 25 μM of ZEA, respectively.

Exposure of porcine pulmonary alveolar macrophages (PAM) to 0.025 ug/ml DON enhanced the phagocytosis of *S. typhimurium* by macrophages by modulating the macrophage cytoskeleton [202]. The phagocytosis of *Mycobacterium avium ssp. Paratuberculosis* (MAP) by BoMacs was also enhanced by OTA [203]; the other *Penicillium* mycotoxins (CIT and MPA) that this group investigated did not show this stimulatory effect on macrophage phagocytosis [203]. 

## 7. In-Vitro Assessment of Efficacy of Risk Mitigation

### 7.1. Strategies to Counteract Mycotoxin Contamination

In an attempt to mitigate the risk of mycotoxin contamination in food and feed, different pre-and post-harvest physical (e.g., crop rotation, thermal treatment, and irradiation), chemical (e.g., acids/bases and absorbents), and biological (e.g., microbial and enzymatic degradation) strategies have been deployed [27,204,205,206,207]. Besides these conventional mitigation methods, nanotechnology may be an innovative solution to mycotoxin contamination [208]. It is not possible in this review to discuss all the approaches; instead, the discussion will focus on remediation strategies that are most widely used in the animal feed industry, especially the use of mycotoxin adsorbents as feed additives.

Among all approaches, the addition of mycotoxin adsorbents to animal feeds, also referred to as “mycotoxin binders”, one of the two classes of mycotoxin detoxifying agents [204], is one of the most widely applied and promising remediation approaches to reduce risk of mycotoxicosis in farm animals [209,210,211,212]. Mycotoxin adsorbents bind mycotoxins in the gastrointestinal tract after the contaminated feed is ingested [207], and the bioavailability of the mycotoxins is reduced by the formation of toxin-adsorbent complexes, which are later excreted in the feces [204]. Mycotoxin absorbents can be classified as either silica-based inorganic compounds, or carbon-based organic polymers [213]. The inorganic absorbents are further sub-grouped into aluminosilicate minerals (clays, including bentonites, montmorillonites, hydrated sodium calcium aluminosilicate, and zeolites), activated charcoal (AC), and synthetic polymers (e.g., cholestyramine). The aluminosilicate minerals are the most widely studied of the silica-based inorganic mycotoxin absorbents [211,213]. The efficacy of inorganic absorbents depends on the physio-chemical structure of both adsorbent and mycotoxin [206,211,214]; this includes the total charge and charge distribution of adsorbents and mycotoxins, adsorbent pore size, and accessible surface area, as well as mycotoxin polarity, solubility, and three-dimensional structure [204,206,211,212]. The efficacy of aluminosilicate adsorbents for reducing aflatoxin B_1_ (AFB_1_) bioavailability is fairly efficient [206], but their binding capacity to other mycotoxins is limited [206,214,215]. In contrast, AC has been reported to effectively bind to DON, ZEA, AFB1, fumonisin B1, and OTA, but it can reduce the absorption of some micronutrients which jeopardize the nutritional value of the feed [204,211,214,216]. Cholestyramine is the most well-known of the synthetic polymers and has been shown to be an effective adsorbent for FB_1_, OTA, and ZEA [210,212,214,217]. Its high cost limits its practical use as a mycotoxin adsorbent [218], and inorganic binders are typically added to feeds at high concentrations to account for their low efficiency [216]. Lastly, since the degradation of bound mycotoxins after they have been excreted is relatively slow, this is another ecological disadvantage of using inorganic adsorbents [207]. 

A commonly used organic adsorbent is yeast cell wall (YCW) from *Saccharomyces cerevisiae* yeast strains [204]. The major functional fractions of YCW responsible for mycotoxin binding include β-D-glucan and α-D-mannan (glucomannan), which bind to mycotoxins via hydrogen bonding and van-der-Waal forces [219,220,221,222,223,224]. The YCW has been shown effective at binding a wide-spectrum of mycotoxins including DON, T-2 toxins, AFB_1_, ZEA, and OTA [207,219,222,223,224,225,226,227,228,229,230,231,232,233]. Heat or acid treatment can further increase the mycotoxin-binding capacity of YCW [229]. Another advantage of YCW products is that they are biodegradable, and therefore the toxin-binder complexes do not accumulate in the environment after being excreted in the feces [219]. The use of lactic acid bacteria (LAB) as an organic dietary mycotoxin-adsorbing agent has recently gained interest [204]. LAB are a group of Gram-positive and non-sporulating bacteria [233], and the strain of LAB that is used to bind to mycotoxins is *Lactobacillus rhamnosus* [204,213]. With glucomannan as the functional component affecting mycotoxin binding capability, the mechanism of LAB is thought to be similar to that of YCW [204]. 

The second class of mycotoxin detoxifying agents is referred to as mycotoxin modifiers. These agents, which include microorganisms and their enzymes, can be applied to reduce the risk of mycotoxicity by biotransforming mycotoxins to less toxic metabolites [204]. Many commercially available mycotoxin detoxifying agents contain a combination of these two classes, capable of both degradation and adsorption. The authors in [234] conducted a study assessing the efficacy of 20 commercial products incubated under aerobic and anaerobic conditions to detoxify DON and ZEA. Their study revealed that only one out of 20 products under anaerobic incubation was effective at completely degrading DON after 24 h and only one tested product completely degraded ZEA under both incubation conditions after 24 h. All the other products incubated under both aerobic and anaerobic conditions showed maximum DON detoxification of only 17%, and only the other four products showed a reduction of ZEN ≥60% [234].

### 7.2. In-Vitro Assessment of Mycotoxin Absorbents

The efficacy of mycotoxin adsorbents has been assessed using both in vitro chemical and cell-based bioassays [231,235,236]. With in-vitro chemical assay, the method involves simulating pH conditions in the gastrointestinal tract of different species during adsorbent-mycotoxin co-incubation, and this is followed by chemical chromatographic analysis such as high-performance liquid chromatography with fluorometric detection [205,230,231,232,234], ultraviolet light detection [237], liquid chromatography-tandem mass spectrometry [238], or gas chromatography [235]. Several adsorption isotherm models have been used following the chromatographic analyses to quantify the adsorption performance of tested adsorbents including the Hill, Langmuir, Freundlich, Brunauer–Emmett–Teller (BET), and non-ideal competitive adsorption (NICA) models [228,230,232]. In a study assessing the binding capacity of various yeast-based products to ZEA, AFB_1,_ and OTA, the most suitable models were the Hill model for ZEA, the Langmuir model for AFB_1,_ and the Freundlich model for OTA [232]. When assessing the binding capacity of YCW products and hydrated sodium calcium aluminosilicate to ZEA, [228] reported that the Hill model was or more suitable than the Freundlich model for evaluating YCW adsorption efficacy, but less suitable for HSCAS (hydrated sodium calcium aluminosilicate) adsorbents. 

In vitro cell-based bioassays have been less utilized for the assessment of mycotoxin adsorbent efficacy. The endpoints of assessment have included cell viability, proliferation, and TEER measurement [231,235,236]. Different cell lines derived from various species and tissues have been used in assessment studies including Caco-2, NIH/3T3-LNCX murine fibroblasts, and MCF-7 human breast cells [231,235]. A study using the differentiated Caco-2 cell line demonstrated that adsorbents such as AC, aluminosilicate minerals, cholestyramine, mannans, and β-glucans exhibited no significant cytotoxicity; however, cholestyramine induced a decrease in cell viability [235]. In the same study, all tested adsorbents except for cholestyramine mitigated the cytotoxic effects of DON, maintaining higher cell viability than even the control [235]. A study using NIH/3T3-LNCX murine fibroblasts also indicated AC showed the highest binding affinity to DON based on cell viability assessment [231]. The study using the MCF-7 cell line has shown that AC and aluminosilicate minerals adsorbents were effective in binding ZEA [231]. Lastly, [236] assessed the binding capacity of a YCW product to *Penicillium* mycotoxins (i.e., CIT, OTA, MPA, patulin and penicillic acid) using a bovine macrophage (BoMacs) cell line, with cell proliferation as a bioassay endpoint. Their results showed that YCW was the most effective in protecting BoMacs cells against OTA, followed by CIT among all five mycotoxins. A study has also shown that illite mineral clay was for protecting AFB1- and OTA-mediated reductions in Caco-2 cell TEER measurements [166].

## 8. Suitability and Limitations of Reviewed Intestinal in Vitro Models

In vitro cell culture models have been extensively used in toxicology, mostly for assessing organ-specific effects of xenobiotics. However, they hardly represent the complexity of the human and animal body [239,240]. The simplicity of in vitro models compared to in vivo however makes it possible to study toxic MOAs in a reproducible manner that may be difficult to be achieved in vivo [140]. In vitro experiments also allow for dose–response analysis of individual mycotoxin exposure as well as their mixtures [8]. The Caco-2, IPEC-1, and IPEC-J2 cell lines reported in the reviewed studies were able to exhibit adequate differentiated intestinal epithelial characteristics, such as proper formation of TJs and polarization in certain culture conditions, immune response-related molecular markers, as well as responsiveness of these characters to mycotoxin exposure with or without risk mitigation methods such as mycotoxin adsorbents; they could represent physiological models of the intestinal epithelial barrier. While these IEC models are used at their undifferentiation status where polarization is not displayed and proper TJs are not formed, they could also be a representation of pathological models of the intestinal epithelial barrier, such as inflammatory bowel diseases (IBD) [241]; they also appear similar to dividing cells in tissue undergoing regeneration or repair after damage [132,134]. Moreover, in terms of assessing the efficacy of mycotoxin adsorbents, with the presence of cells, the in-vitro cell culture could also detect unpredictable tenside-like activities of adsorbents that could affect cell membrane permeability and result in an increase in cellular uptake and toxicity of mycotoxins [242]. 

Although in vitro models are useful tools and provide valuable information, results should be interpreted with care as there are some limitations with these in vitro models. First, cell lines lack cellular diversity in the single-cell type system. For example, the Caco-2 cell line is not able to differentiate into goblet cells that are present in vivo, thus, mucins and mucus, which are present under normal physiological conditions, are lacking in vitro [140,243]. Second, in vitro cell models may lack certain phenotypes and characteristics that are exhibited in vivo [140,243,244,245,246,247,248]. For example, the HT-29 human colon cancer cell line cannot form proper TJs under certain growth conditions, whereas the T84 human colon cancer cell line is an excellent model to examine epithelial barrier function due to its high TEER properties [139]. Also, the TEER of Caco-2 cells was reported to be smaller than in vivo [249], and neither Caco-2, T84 nor IPEC-J2 cells express claudin-2 [250]. Third, cell culture conditions, such as passage number, media formulation, and culture time, can also affect the conditions of cell lines [139,251]. Other limitations include a lack of relevant factors occurring in vivo, immortalization, limited survival, and metabolic imbalance [231,252].

To date, limited studies have investigated the effects of mycotoxins on the intestinal barrier functions using 2D co-culture models. However, an IEC + immune cell co-culture system may be more appropriate than monocultures to study the effects of mycotoxins on the intestinal barrier function because 2D co-culture models better represent the epithelial structure and function in vivo. This 2D system enables the study of cell–cell interactions by both direct cell contact and soluble factors that are secreted between IECs and immune cells, depending on the co-culture set-up [129,253]. However, 2D co-culture models do have some limitations. Compared to 3D co-culture models, 2D co-culture models have reduced cell–cell interactions, lack cell–matrix interactions, and may be lacking in complete tissue architecture [254]. When compared to monoculture models, a limitation of 2D co-culture is that a wider range of variables could affect the outcomes of co-culture models including cell culture conditions, the size and ratio of different cultured cell populations, and time scale of the experiments with the interactions between populations considered [255]. 

## 9. Conclusion and Discussion

Mycotoxins present an issue worldwide due to their ability to contaminate agricultural commodities and to pose a health risk to both humans and animals that have ingested the contaminated food and feed. Climate change will likely favor more mycotoxin contamination [2,7]. Since mycotoxins are commonly present as co-contaminants, it is not only important to understand their MOAs, many of which are unknown, but also to understand how they interact with each other to affect exposed humans and animals.

Since the intestine is the major site of mycotoxin interaction following oral exposure, understanding these interactions at the intestinal level is critical for risk assessment and mitigation. The intestine functions as a semi-permeable physical and immunological barrier and is the major site of nutrient absorption. Therefore, any adverse effects mycotoxins pose to the intestine, such as changes in intestinal permeability, cytokine production, and cell viability may be a constraint to animal health and production.

In vitro cell culture models of the intestinal barrier have been used to mimic oral exposure to mycotoxins. These intestinal models are usually based on a monolayer epithelial cell culture system, sometimes grown on membrane inserts to better mimic the intestinal barrier for assessing the intestinal transport of mycotoxins, the impact of different routes of exposure, and how mycotoxin exposure impacts the translocation of pathogens [143,148,152,256]. 

A number of different mitigation approaches, including the use of mycotoxin adsorbents, have been developed and applied to help reduce the adverse effects of mycotoxins on animals, but their efficacy varies depending on physio-chemical properties of both adsorbents and mycotoxins. Given this, there is an ongoing need for the development of novel more effective mycotoxin adsorbents and for their efficacy assessment. Given that in-vitro cell culture can help to better understand what actually happens at the intestinal level [235], the possible cytotoxic effects of mycotoxin adsorbents on the gut epithelium and their mycotoxin binding efficacy can be assessed using in-vitro cell-based bioassays based on functional parameters such as cell viability and TEER values [231,235].

Most of the cell culture studies collated in this review are based on the in vitro monoculture system. However, it may be more appropriate and efficient to co-culture various cell types, such as the IEC + macrophage co-culture model, to simulate a more complex in-vitro system that better reflects the intestinal mucosa physiologically and morphologically. Although there are limitations associated with cell culture models, in vitro monoculture, or even better a co-culture system, is an efficient approach for initial toxicity assessment of mycotoxins and their mixtures and assessment of adsorbent efficacy. It is also an efficient approach for determining the MOAs for both individual and combined mycotoxins that exhibit species- and organ-specific toxicity at the cellular and molecular level.

## 10. Suggestions for Future Research

Although in vitro and in vivo toxicity data for DON is abundant, toxicity data for other mycotoxins is limited, especially with regards to *Penicillium* mycotoxins, which are commonly detected in forage, particularly silage [257,258]. There is also a lack of in vitro and in vivo toxicity data concerning the combined toxic effects of mycotoxins. Moreover, exposure guidelines throughout the world are all based on individual mycotoxins, and multi-exposure has raised a question about the health risk of co-occurring mycotoxins. As in most cases, feed and food can be contaminated with multiple mycotoxins and the combined toxicity of mycotoxin mixtures cannot always be predicted based on their individual toxicity [5]. Thus, more studies should investigate the effects of multi-mycotoxin exposure to provide guidance for toxicological evaluation and reflect on the suitability of current mycotoxin exposure guidelines.

In vitro and in vivo mycotoxin toxicity studies have focused more on monogastric animals over ruminants, as ruminants are considered more resistant to mycotoxins due to the ability of rumen microbes to detoxify mycotoxins into non-toxic compounds [259]. However, the safety of ruminant species should be more thoroughly considered. Certain mycotoxins with antimicrobial properties, for example, can impair the function of the rumen and intestinal microflora, thus, decreasing their capacity to degrade mycotoxin [25,27]. Moreover, ruminant animals in certain production stages are more susceptible to mycotoxins. For example, ruminants in the transition period have a negative energy balance and are particularly sensitive to mycotoxin contamination in feed [25]. Also, newly-weaned ruminants can be prone to mycotoxin exposure because the rumen microbiota is not fully established or functional to protect young ruminants from mycotoxins [260]. Some mycotoxins may by-pass the rumen intact instead of being detoxified in the rumen [79].

In vitro cell culture systems (monoculture or co-culture systems) could be a useful and effective approach to start with for studying organ- and species-specific complicated issues of mycotoxin toxicity at the cellular and molecular levels such as interactions between different mycotoxins [151], comparative toxicity of mycotoxins, and their metabolites [155]. Moreover, cell culture systems could be appropriate methods to study biotransformation of mycotoxins in animal cells, for example, the cell models could express certain enzymes such as Cytochromes P450 (CYPs) that might interact with tested mycotoxins by biotransforming them to the resulting metabolites [16,261]. 

## Figures and Tables

**Table 1:** Summary of effects of individual mycotoxins on intestinal epithelial cell (IEC) viability.

Mycotoxin	IEC model	Exposure Duration	Tested Exposure Concentration	Cytotoxicity Assay	LC50/Effective Concentration (ECs)	References
**DON**	Caco-2 (differentiated)	24 h	0, 1.39, 4.17, 12.5, 37.5 μM	LDH release	EC: 37.5 μM	[152]
	Caco-2	48 h	0, 0.05, 0.1, 0.3, 0.5, 1, 3, 5, 10 μM	Luminescent Cell Viability Assay	LC50 =1.3 uM; EC: 0.5–10μM	[153]
	Caco-2	24 h	0, 0.25, 1, 2.5, 5, 10 μM	MTS Assay	LC50 = 10 μM	[154]
				Neutral Red	LC50 = 3.7 μM	
		72 h	0, 0.25, 1, 2.5, 5, 10 μM	MTS Assay	LC50 = 4.3 μM	
				Neutral Red	LC50 = 3.7 μM	
	Caco-2 (differentiated)	24 h	0, 0.25, 1, 2.5, 5, 10 μM	MTS Assay	LC50 > 10 μM	[154]
				Neutral Red	LC50 > 10 μM	
		72 h	0, 0.25, 1, 2.5, 5, 10 μM	MTS Assay	LC50 > 10 μM	
				Neutral Red	LC50 > 10 μM	
	Caco-2	72 h	1–150 μM	Neutral Red	LC50 = 21.5 μM; EC: 10 μM	[155]
				MTT Assay	LC50 = 25 μM; EC: 10 μM	
	Caco-2	24 h	0, 0.001, 0.01, 0.1, 1, 10, 25, 50, 100 μM	CCK-8	LC50 = 21.94 μM	[156]
		48 h			LC50 = 9.39 μM	
		72 h			LC50 = 6.18 μM	
	IPEC-1	24 h	0, 0.34, 0.67, 1.7, 3.4, 6.7 10.2, 13.4 μM	MTT Assay	EC: 1.7, 3.4, 10.2, 13.4 μM	[143]
		48 h			EC: 0.34 μM; 1.7–13.4 μM	
		72 h			EC: 0.34 μM; 1.7- 13.4 μM	
	IPEC-1 (in serum-free media)	24 h	0, 0.67, 6.7 μM	LDH release	NA	
		48 h			EC: 6.7 μM	
		72 h			EC: 6.7 μM	
	IPEC-1 (in complete media)	24 h	0, 0.67, 6.7 μM	Neutral Red	EC: 0.67, 6.7 μM	
		48 h			EC: 6.7 μM	
		72 h			EC: 6.7 μM	
	IPEC-1 (in serum-free media)	24 h	0, 0.67, 6.7 μM	Neutral Red	EC: 0.67, 6.7 μM	
		48 h			EC: 6.7 μM	
		72 h			EC: 6.7 μM	
	IPEC-J2	24 h	0, 0.34, 0.67, 1.7, 3.4, 6.7 10.2, 13.4 μM	MTT Assay	EC: 0.34, 3.4, 6.7, 13.4 μM	[143]
		48 h			EC: 1.7–13.4 μM	
		72 h			EC: 1.7–13.4 μM	
		14 d	0, 0.17, 0.34, 0.67, 1.02, 1.34, 1.7 μM		0.67, 1.02, 1.34, 1.7 μM	
	IPEC-J2	24 h	0, 0.67, 6.7 μM	LDH release	NA	[143]
		48 h			EC: 6.7 μM	
		72 h			NA	
	IPEC-J2	24 h	0, 0.67, 6.7 μM	Neutral Red	EC: 0.67, 6.7 μM	[143]
		48 h			EC: 6.7 μM	
		72 h			EC: 6.7 μM	
	IPEC-J2 (in serum-free media)	24 h	0, 0.67, 6.7 μM	Neutral Red	EC: 0.67, 6.7 μM	[143]
		48 h			EC: 6.7 μM	
		72 h			EC: 6.7 μM	
	IPEC-J2 (basolateral)	24 h	0, 0.67, 1.7, 6.7, 13.4 μM	DAPI staining	NA	[157]
		48 h			EC: 6.7, 13.4 μM	
		72 h			EC: 6.7, 13.4 μM	
	IPEC-J2	24 h	0, 0.034, 0.085, 0.17, 0.34, 0.85, 1.7, 3.4, 17, 34 μM	Neutral Red	EC: 0.85–34 μM	[132]
	IPEC-J2	72 h	0, 3.4, 8.5, 17, 25.5, 34 μM	Annexin-V-FITC/ PI	EC: 8.5–34 μM	[158]
	IPEC-J2	72 h	0, 3.4, 17, 34, 51, 67 μM	Annexin-V-FITC/ PI	LC50 = 10.47 μM	[159]
	IPEC-J2 (differentiated)	72 h	0, 3.4, 17, 34, 51, 67 μM	Annexin-V-FITC/ PI	LC50 = 46.9 μM	[159]
	IPEC-J2	6 h	0, 0.67, 6.7 μM	CCK-8 Assay	EC: 0.67, 6.7 μM	[160]
		12 h				
		24 h				
		48 h				
		72 h				
	IPEC-J2	48 h	0, 0.25, 0.5, 1, 2 μM	MTT Assay	LC50 = 1.83μM; EC:1–2 μM	[161]
	IPEC-J2	24 h	0, 0.43, 0.85, 1.7, 3.4, 6.7 μM	CCK-8 Assay	EC: 0.85–6.7 μM	[162]
**ZEA**	Caco-2	72 h	1–150 μM	Neutral Red	LC50 = 15 μM	[155]
				MTT Assay	LC50 = 25 μM	
	Caco-2	24 h	0, 0.001, 0.01, 0.1, 1, 10, 25, 50, 100 μM	CCK-8	LC50 = 62.67 μM	[156]
		48 h			LC50 = 56.96 μM	
		72 h			LC50 = 34.36 μM	
	IPEC-1	24 h	0, 0.1, 1, 10, 100 μM	XTT Assay	EC: 100 μM	[163]
	IPEC-1	24 h	0, 0.1, 1, 10, 100 μM	XTT Assay	EC: 100 μM	[164]
			0, 0.1, 1, 10, 100 μM	Neutral Red	EC: 100 μM	
	IPEC-J2	48 h	0, 5, 10, 20, 40 μM	MTT Assay	EC: 10, 40 μM	[161]
	IPEC-J2	48 h	0, 15.5, 31, 62, 124, 248 μM	MTT Assay	LC50 = 62.1 μM; EC: 62–248 μM	[46]
	IPEC-J2	72 h	0, 19.9, 39.8, 44.73, 59.7, 79.6, 99.5 μM	Annexin-V-FITC/PI	EC: 44.73–99.5 μM	[158]
	Caco-2	24 h	0, 0.032, 0.16, 0.32, 1.6, 3.2 μM	MTT Assay	EC: 3.2 μM	[165]
**AFB1**		48 h			EC: 0.32—3.2 μM	
		72 h			EC: 1.6–3.2 μM	
	Caco-2 (differentiated)	24 h	0, 0.032, 0.16, 0.32, 1.6, 3.2 μM	MTT Assay	EC: 1.6 μM	[165]
		48 h			EC: 1.6–3.2 μM	
		72 h			EC: 0.16–3.2 μM	
	Caco-2	24 h	0, 0.032, 0.16, 0.32, 1.6, 3.2 μM	LDH release	EC: 1.6–3.2 μM	[165]
		48 h			EC: 0.32–3.2 μM	
		72 h			EC: 0.32–3.2 μM	
	Caco-2 (differentiated)	24 h	0, 0.032, 0.16, 0.32, 1.6, 3.2 μM	LDH release	EC: 3.2 μM	[165]
		48 h			EC: 0.032–3.2 μM	
		72 h			EC: 0.032–3.2 μM	
	Caco-2	24 h	0, 1,3, 10, 30, 100 μM	MTT Assay	LC50 = 5.39 μM EC: 1–100 μM	[166]
				LDH release	LC50 = 10 μM EC: 3–100 μM	
	Caco-2	24 h	0–100 μM	Neutral Red	LC50 = 10 μM	[167]
		48 h			LC50 = 2 μM	
		72 h			LC50 = 0.75 μM	
**CIT**	Caco-2	48 h	0, 399.6, 999 μM	Crystal Violet staining (CVS)	EC: 399.6, 999 μM	[168]
	HCT116	36 h	0, 75, 150, 300 μM	Fluorescein diacetate (FDA) staining	LC50 = 300 μM; EC: 150- 300 μM	[169]
**MPA**	Caco-2	48 h	0, 0.0078, 0.078, 0.78, 7.8, 78, 780 μM	MTS Assay	LC50 > 780 uM	[75]
	Caco-2 (differentiated)					
**OTA**	Caco-2	24 h	0, 1, 3, 10, 30, 100 μM	MTT Assay	LC50 = 21.25 μM; EC: 1–100 μM	[166]
				LDH release	LC50 = 16.85 μM; EC: 1–100 μM	
	Caco-2	24 h	1–200 μM	MTT Assay	LC50 = 145.36 μM	[170]

**Table 2:** Summary of effects of selected mycotoxins on tight junction gene and protein expression.

Mycotoxins	IEC model	Exposure Duration	Exposure Concentration	Effects of Selected Mycotoxins on Gene and Protein Expression of TJs		References
				Gene Expression	Protein Expression	
DON	Caco-2	24 h	0, 1.39, 4.17, 12.5 μM	Increase in CLDN1, CLDN3, CLDN4, OCLN, ZO-1	Decrease in CLDN1, CLDN3, CLDN4	[152]
	Caco-2	24 h	0, 0.17, 1.7, 17 μM	Increase in CLDN4, OCLN	Decrease in CLDN4	[183]
	Caco-2	48 h	0, 5, 10, 20, 50, 100 μM	N/A	Decrease in CLDN4	[184]
	IPEC-1	48 h	0, 5, 10, 20, 50 μM	N/A	Decrease CLDN3, CLDN4	[184]
	IPEC-1	48 h	0.67, 6.7 μM	N/A	Decrease in ZO-1	[143]
	IPEC-J2	48 h	0.67, 6.7 μM	N/A	Decrease in ZO-1	[143]
	IPEC-J2	12 h	0, 4 μM	Decrease in CLDN3; increase CLDN4, OCLN, ZO-1	Decrease CLDN3, CLDN4	[189]
AFB1	Caco-2	7 days	0, 1,3, 10, 30 μM	Decrease in CLDN3, OCLN	N/A	[166]
OTA	Caco-2	24 h	0, 100 μM	N/A	Decrease in CLDN3 and CLDN4	[185]
	Caco-2	24 h	0, 100 μM	N/A	Decrease in CLDN3 and CLDN4	[192]
	Caco-2	7 days	1, 3, 10, 30 μM	Decrease in CLDN3, CLDN4 and OCLN	N/A	[166]

## References

[1] Mohammadi H. (2011). A Review of Aflatoxin M1, Milk, and Milk Products. Aflatoxins–Biochemistry and Molecular Biology.

[2] Bryden W.L. (2012). Mycotoxin contamination of the feed supply chain: Implications for animal productivity and feed security. Anim. Feed Sci. Technol..

[3] Marin S., Ramos A.J., Cano-Sancho G., Sanchis V. (2013). Mycotoxins: Occurrence, toxicology, and exposure assessment. Food Chem. Toxicol..

[4] Völkel I., Schröer-Merker E., Czerny C.-P. (2011). The Carry-Over of Mycotoxins in Products of Animal Origin with Special Regard to Its Implications for the European Food Safety Legislation. Food Nutr. Sci..

[5] Smith M.-C., Madec S., Coton E., Hymery N. (2016). Natural Co-Occurrence of Mycotoxins in Foods and Feeds and Their in vitro Combined Toxicological Effects. Toxins.

[6] Council for Agricultural Science and Technology (2003). Mycotoxins: Risks in Plant, Animal, and Human Systems.

[7] Lee H.-S., Kwon N.J., Kim Y., Lee H. (2018). Prediction of mycotoxin risks due to climate change in Korea. Appl. Biol. Chem..

[8] Alassane-Kpembi I., Puel O., Oswald I.P. (2015). Toxicological interactions between the mycotoxins deoxynivalenol, nivalenol and their acetylated derivatives in intestinal epithelial cells. Arch. Toxicol..

[9] Grenier B., Applegate T. (2013). Modulation of Intestinal Functions Following Mycotoxin Ingestion: Meta-Analysis of Published Experiments in Animals. Toxins.

[10] Bouhet S., Oswald I.P. (2005). The effects of mycotoxins, fungal food contaminants, on the intestinal epithelial cell-derived innate immune response. Vet. Immunol. Immunopathol..

[11] Bain C.C., Mowat A.M. (2014). Macrophages in intestinal homeostasis and inflammation. Immunol. Rev..

[12] Bain C.C., Scott C.L., Uronen-Hansson H., Gudjonsson S., Jansson O., Grip O., Guilliams M., Malissen B., Agace W.W., Mowat A.M. (2013). Resident and pro-inflammatory macrophages in the colon represent alternative context-dependent fates of the same Ly6C hi monocyte precursors. Mucosal Immunol..

[13] Pelaseyed T., Bergström J.H., Gustafsson J.K., Ermund A., Birchenough G.M.H., Schütte A., Van der Post S., Svensson F., Rodríguez-Piñeiro A.M., Nyström E.E.L. (2014). The mucus and mucins of the goblet cells and enterocytes provide the first defense line of the gastrointestinal tract and interact with the immune system. Immunol. Rev..

[14] Stadnyk A.W. (2002). Intestinal Epithelial Cells as a Source of Inflammatory Cytokines and Chemokines. Can. J. Gastroenterol..

[15] Swamy M., Jamora C., Havran W., Hayday A. (2010). Epithelial decision makers: In search of the “epimmunome”. Nat. Immunol..

[16] Sergent T., Ribonnet L., Kolosova A., Garsou S., Schaut A., De Saeger S., Van Peteghem C., Larondelle Y., Pussemier L., Schneider Y.-J. (2008). Molecular and cellular effects of food contaminants and secondary plant components and their plausible interactions at the intestinal level. Food Chem. Toxicol..

[17] Prelusky D.B., Trenholm H.L., Rotter B.A., Miller J.D., Savard M.E., Yeung J.M., Scott P.M., Jackson L.S., DeVries J.W., Bullerman L.B. (1996). Biological Fate of Fumonisin B1 in Food-Producing Animals. Fumonisins in Food.

[18] Shephard G.S., Thiel P.G., Sydenham E.W., Savard M.E. (1995). Fate of a single dose of 14C-labelled fumonisin B1 in vervet monkeys. Nat. Toxins.

[19] Biehl M.L., Prelusky D.B., Koritz G.D., Hartin K.E., Buck W.B., Trenholm H.L. (1993). Biliary Excretion and Enterohepatic Cycling of Zearalenone in Immature Pigs. Toxicol. Appl. Pharmacol..

[20] Roth A., Chakor K., EkuéCreepy E., Kane A., Roschenthaler R., Dirheimer G. (1988). Evidence for an enterohepatic circulation of ochratoxin A in mice. Toxicology.

[21] De Angelis I., Friggè G., Raimondi F., Stammati A., Zucco F., Caloni F. (2005). Absorption of Fumonisin B1 and aminopentol on an in vitro model of intestinal epithelium; the role of P-glycoprotein. Toxicon.

[22] Creppy E.E. (2002). Update of survey, regulation and toxic effects of mycotoxins in Europe. Toxicol. Lett..

[23] Taranu I., Marin D.E., Pistol G.C., Motiu M., Pelinescu D. (2015). Induction of pro-inflammatory gene expression by Escherichia coli and mycotoxin zearalenone contamination and protection by a Lactobacillus mixture in porcine IPEC-1 cells. Toxicon.

[24] Liew W.-P.-P., Mohd-Redzwan S. (2018). Mycotoxin: Its Impact on Gut Health and Microbiota. Front. Cell. Infect. Microbiol..

[25] Fink-Gremmels J. (2008). The role of mycotoxins in the health and performance of dairy cows. Vet. J..

[26] Maresca M., Fantini J. (2010). Some food-associated mycotoxins as potential risk factors in humans predisposed to chronic intestinal inflammatory diseases. Toxicon.

[27] Wambacq E., Vanhoutte I., Audenaert K., Gelder L.D., Haesaert G. (2016). Occurrence, prevention and remediation of toxigenic fungi and mycotoxins in silage: A review. J. Sci. Food Agric..

[28] Kashyap P.C., Marcobal A., Ursell L.K., Larauche M., Duboc H., Earle K.A., Sonnenburg E.D., Ferreyra J.A., Higginbottom S.K., Million M. (2013). Complex Interactions Among Diet, Gastrointestinal Transit, and Gut Microbiota in Humanized Mice. Gastroenterology.

[29] Patel R.M., Lin P.W. (2010). Developmental biology of gut-probiotic interaction. Gut Microbes.

[30] Yano J.M., Yu K., Donaldson G.P., Shastri G.G., Ann P., Ma L., Nagler C.R., Ismagilov R.F., Mazmanian S.K., Hsiao E.Y. (2015). Indigenous Bacteria from the Gut Microbiota Regulate Host Serotonin Biosynthesis. Cell.

[31] Karlovsky P., Suman M., Berthiller F., De Meester J., Eisenbrand G., Perrin I., Oswald I.P., Speijers G., Chiodini A., Recker T. (2016). Impact of food processing and detoxification treatments on mycotoxin contamination. Mycotoxin Res..

[32] Mannaa M., Kim K.D. (2017). Influence of Temperature and Water Activity on Deleterious Fungi and Mycotoxin Production during Grain Storage. Mycobiology.

[33] Tola M., Kebede B. (2016). Occurrence, importance and control of mycotoxins: A review. Cogent Food Agric..

[34] D’Mello J.P.F., Placinta C.M., Macdonald A.M.C. (1999). Fusarium mycotoxins: A review of global implications for animal health, welfare and productivity. Anim. Feed Sci. Technol..

[35] Scheidegger K., Payne G. (2003). Unlocking the Secrets Behind Secondary Metabolism: A Review of Aspergillus flavus from Pathogenicity to Functional Genomics. J. Toxicol. Toxin Rev..

[36] Doughari J. (2015). The Occurrence, Properties and Significance of Citrinin Mycotoxin. J. Plant Pathol. Microbiol..

[37] Schneweis I., Meyer K., Hormansdorfer S., Bauer J. (2000). Mycophenolic Acid in Silage. Appl. Environ. Microbiol..

[38] Bennett J.W., Klich M. (2003). Mycotoxins. Clin. Microbiol. Rev..

[39] Pestka J.J. (2010). Deoxynivalenol: Mechanisms of action, human exposure, and toxicological relevance. Arch. Toxicol..

[40] Maresca M. (2013). From the Gut to the Brain: Journey and Pathophysiological Effects of the Food-Associated Trichothecene Mycotoxin Deoxynivalenol. Toxins Basel.

[41] Sobrova P., Adam V., Vasatkova A., Beklova M., Zeman L., Kizek R. (2010). Deoxynivalenol and its toxicity. Interdiscip. Toxicol..

[42] Pestka J.J. (2007). Deoxynivalenol: Toxicity, mechanisms and animal health risks. Anim. Feed Sci. Technol..

[43] Rocha O., Ansari K., Doohan F.M. (2005). Effects of trichothecene mycotoxins on eukaryotic cells: A review. Food Addit. Contam..

[44] Marin D.E., Taranu I., Burlacu R., Manda G., Motiu M., Neagoe I., Dragomir C., Stancu M., Calin L. (2011). Effects of zearalenone and its derivatives on porcine immune response. Toxicol. Vitr..

[45] Abid-Essefi S., Ouanes Z., Hassen W., Baudrimont I., Creppy E., Bacha H. (2004). Cytotoxicity, inhibition of DNA and protein syntheses and oxidative damage in cultured cells exposed to zearalenone. Toxicol. Vitr..

[46] Fan W., Shen T., Ding Q., Lv Y., Li L., Huang K., Yan L., Song S. (2017). Zearalenone induces ROS-mediated mitochondrial damage in porcine IPEC-J2 cells. J. Biochem. Mol. Toxicol..

[47] Hassen W., Ayed-Boussema I., Oscoz A.A., De Cerain Lopez A., Bacha H. (2007). The role of oxidative stress in zearalenone-mediated toxicity in Hep G2 cells: Oxidative DNA damage, gluthatione depletion and stress proteins induction. Toxicology.

[48] Liu M., Gao R., Meng Q., Zhang Y., Bi C., Shan A. (2014). Toxic Effects of Maternal Zearalenone Exposure on Intestinal Oxidative Stress, Barrier Function, Immunological and Morphological Changes in Rats. PLoS ONE.

[49] Zinedine A., Soriano J.M., Moltó J.C., Mañes J. (2007). Review on the toxicity, occurrence, metabolism, detoxification, regulations and intake of zearalenone: An oestrogenic mycotoxin. Food Chem. Toxicol..

[50] Alshannaq A., Yu J.-H. (2017). Occurrence, Toxicity, and Analysis of Major Mycotoxins in Food. Int. J. Environ. Res. Public. Health.

[51] Rushing B.R., Selim M.I. (2019). Aflatoxin B1: A review on metabolism, toxicity, occurrence in food, occupational exposure, and detoxification methods. Food Chem. Toxicol..

[52] IARC, International Agency for Research on Cancer, Weltgesundheitsorganisation (2012). Monographs on the Evaluation of Carcinogenic Risks to Humans, Volume 100 F, Chemical Agents and Related Occupations: This Publication Represents the Views and Expert Opinions of an IARC Working Group on the Evaluation of Carcinogenic Risks to Humans, which Met in Lyon, 20–27 October 2009.

[53] Amstad P., Levy A., Emerit I., Cerutti P. (1984). Evidence for membrane-mediated chromosomal damage by aflatoxin B _1_ in human lymphocytes. Carcinogenesis.

[54] Bouslimi A., Bouaziz C., Ayed-Boussema I., Hassen W., Bacha H. (2008). Individual and combined effects of ochratoxin A and citrinin on viability and DNA fragmentation in cultured Vero cells and on chromosome aberrations in mice bone marrow cells. Toxicology.

[55] Liu B.-H., Yu F.-Y., Wu T.-S., Li S.-Y., Su M.-C., Wang M.-C., Shih S.-M. (2003). Evaluation of genotoxic risk and oxidative DNA damage in mammalian cells exposed to mycotoxins, patulin and citrinin. Toxicol. Appl. Pharmacol..

[56] Klaric M.S., Zeljezic D., Domijan A.-M., Peraica M., Pepeljnjak S. (2007). Cytotoxicity, genotoxicity and apoptosis induced by ochratoxin A and citrinin in porcine kidney PK15 cells: Effects of single and combined mycotoxins. Toxicol. Lett..

[57] Heussner A.H., Bingle L.E.H. (2015). Comparative Ochratoxin Toxicity: A Review of the Available Data. Toxins.

[58] Marquardt R.R., Frohlich A.A. (1992). A Review of Recent Advances in Understanding Ochratoxicosis’l2. J. Anim. Sci..

[59] Peraica M., Domijan A.-M., Matašin M., Lucić A., Radić B., Delaš F., Horvat M., Bosanac I., Balija M., Grgičević D. (2001). Variations of ochratoxin A concentration in the blood of healthy populations in some Croatian cities. Arch. Toxicol..

[60] Fink-Gremmels J. (2005). Conclusions from the workshops on Ochratoxin A in Food: Recent developments and significance, organized by ILSI Europe in Baden (Austria), 29 June–1 July 2005. Food Addit. Contam..

[61] Grollman A.P., Jelaković B. (2007). Role of Environmental Toxins in Endemic (Balkan) Nephropathy. J. Am. Soc. Nephrol..

[62] Akbari P., Braber S., Varasteh S., Alizadeh A., Garssen J., Fink-Gremmels J. (2017). The intestinal barrier as an emerging target in the toxicological assessment of mycotoxins. Arch. Toxicol..

[63] Kuiper-Goodman T., Scott P.M. (1989). Risk assessment of the mycotoxin ochratoxin A. Biomed. Environ. Sci. BES.

[64] Adler M., Müller K., Rached E., Dekant W., Mally A. (2009). Modulation of key regulators of mitosis linked to chromosomal instability is an early event in ochratoxin A carcinogenicity. Carcinogenesis.

[65] Cui J., Xing L., Li Z., Wu S., Wang J., Liu J., Wang J., Yan X., Zhang X. (2010). Ochratoxin A induces G2 phase arrest in human gastric epithelium GES-1 cells in vitro. Toxicol. Lett..

[66] Czakai K., Müller K., Mosesso P., Pepe G., Schulze M., Gohla A., Patnaik D., Dekant W., Higgins J.M.G., Mally A. (2011). Perturbation of Mitosis through Inhibition of Histone Acetyltransferases: The Key to Ochratoxin A Toxicity and Carcinogenicity?. Toxicol. Sci..

[67] Mally A. (2012). Ochratoxin A and Mitotic Disruption: Mode of Action Analysis of Renal Tumor Formation by Ochratoxin A. Toxicol. Sci..

[68] Mally A., Dekant W. (2009). Mycotoxins and the kidney: Modes of action for renal tumor formation by ochratoxin A in rodents. Mol. Nutr. Food Res..

[69] Rached E., Pfeiffer E., Dekant W., Mally A. (2006). Ochratoxin A: Apoptosis and Aberrant Exit from Mitosis due to Perturbation of Microtubule Dynamics?. Toxicol. Sci..

[70] Wang Y., Liu J., Cui J., Xing L., Wang J., Yan X., Zhang X. (2012). ERK and p38 MAPK signaling pathways are involved in ochratoxin A-induced G2 phase arrest in human gastric epithelium cells. Toxicol. Lett..

[71] Pfohl-Leszkowicz A., Manderville R.A. (2012). An Update on Direct Genotoxicity as a Molecular Mechanism of Ochratoxin A Carcinogenicity. Chem. Res. Toxicol..

[72] Sorrenti V., Di Giacomo C., Acquaviva R., Barbagallo I., Bognanno M., Galvano F. (2013). Toxicity of Ochratoxin A and Its Modulation by Antioxidants: A Review. Toxins.

[73] Boysen M.E., Jacobsson K.-G., Schnürer J. (2000). Molecular Identification of Species from the Penicillium roqueforti Group Associated with Spoiled Animal Feed. Appl. Environ. Microbiol..

[74] Driehuis F. (2013). Silage and the safety and quality of dairy foods: A review. Agric. Food Sci..

[75] Hymery N., Mounier J., Coton E. (2018). Effect of Penicillium roqueforti mycotoxins on Caco-2 cells: Acute and chronic exposure. Toxicol. Vitr..

[76] Pereyra C., Alonso V., Rosa C., Chiacchiera S., Dalcero A., Cavaglieri L. (2008). Gliotoxin natural incidence and toxigenicity of *Aspergillus fumigatus* isolated from corn silage and ready dairy cattle feed. World Mycotoxin J..

[77] Richard E., Heutte N., Sage L., Pottier D., Bouchart V., Lebailly P., Garon D. (2007). Toxigenic fungi and mycotoxins in mature corn silage. Food Chem. Toxicol..

[78] Storm I.M.L.D., Kristensen N.B., Raun B.M.L., Smedsgaard J., Thrane U. (2010). Dynamics in the microbiology of maize silage during whole-season storage. J. Appl. Microbiol..

[79] Gallo A., Giuberti G., Frisvad J.C., Bertuzzi T., Nielsen K.F. (2015). Review on Mycotoxin Issues in Ruminants: Occurrence in Forages, Effects of Mycotoxin Ingestion on Health Status and Animal Performance and Practical Strategies to Counteract Their Negative Effects. Toxins.

[80] Bentley R. (2000). Mycophenolic Acid:  A One Hundred Year Odyssey from Antibiotic to Immunosuppressant. Chem. Rev..

[81] Eugui E.M., Almquist S.J., Muller C.D., Allison A.C. (1991). Lymphocyte-Selective Cytostatic and Immunosuppressive Effects of Mycophenolic Acid in vitro: Role of Deoxyguanosine Nucleotide Depletion. Scand. J. Immunol..

[82] Cole R.J., Cox R.H. (1981). Handbook of Toxic Fungal Metabolites.

[83] Puel O., Tadrist S., Galtier P., Oswald I.P., Delaforge M. (2005). Byssochlamys nivea as a Source of Mycophenolic Acid. Appl. Environ. Microbiol..

[84] Dzidic A., Meyer H.H.D., Bauer J., Pfaffl M.W. (2010). Long-term effects of mycophenolic acid on the immunoglobulin and inflammatory marker-gene expression in sheep white blood cells. Mycotoxin Res..

[85] Swanson S.P., Nicoletti J., Rood H.D., Buck W.B., Cote L.M., Yoshizawa T. (1987). Metabolism of three trichothecene ycotoxins, T-2 toxin, diacetoxyscirpenol and deoxynivalenol, by bovne rumen microorganisms. J. Chromatogr. B. Biomed. Sci. Appl..

[86] Cummings J.H., Antoine J.-M., Azpiroz F., Bourdet-Sicard R., Brandtzaeg P., Calder P.C., Gibson G.R., Guarner F., Isolauri E., Pannemans D. (2004). PASSCLAIM^1^–Gut health and immunity. Eur. J. Nutr..

[87] Brandtzaeg P. (2011). The gut as communicator between environment and host: Immunological consequences. Eur. J. Pharmacol..

[88] Vancamelbeke M., Vermeire S. (2017). The intestinal barrier: A fundamental role in health and disease. Expert Rev. Gastroenterol. Hepatol..

[89] Bischoff S.C., Barbara G., Buurman W., Ockhuizen T., Schulzke J.-D., Serino M., Tilg H., Watson A., Wells J.M. (2014). Intestinal permeability—A new target for disease prevention and therapy. BMC Gastroenterol..

[90] Sato T., Vries R.G., Snippert H.J., Van de Wetering M., Barker N., Stange D.E., Van Es J.H., Abo A., Kujala P., Peters P.J. (2009). Single Lgr5 stem cells build crypt-villus structures in vitro without a mesenchymal niche. Nature.

[91] Mueller C., Macpherson A.J. (2006). Layers of mutualism with commensal bacteria protect us from intestinal inflammation. Gut.

[92] Groschwitz K.R., Hogan S.P. (2009). Intestinal Barrier Function: Molecular Regulation and Disease Pathogenesis. J. Allergy Clin. Immunol..

[93] Peterson L.W., Artis D. (2014). Intestinal epithelial cells: Regulators of barrier function and immune homeostasis. Nat. Rev. Immunol..

[94] Donaldson G.P., Lee S.M., Mazmanian S.K. (2016). Gut biogeography of the bacterial microbiota. Nat. Rev. Microbiol..

[95] Ley R.E., Peterson D.A., Gordon J.I. (2006). Ecological and Evolutionary Forces Shaping Microbial Diversity in the Human Intestine. Cell.

[96] Schenk M., Mueller C. (2008). The mucosal immune system at the gastrointestinal barrier. Best Pract. Res. Clin. Gastroenterol..

[97] Mukherjee S., Hooper L.V. (2015). Antimicrobial Defense of the Intestine. Immunity.

[98] Gill N., Wlodarska M., Finlay B.B. (2010). The future of mucosal immunology: Studying an integrated system-wide organ. Nat. Immunol..

[99] Philpott D.J., Sorbara M.T., Robertson S.J., Croitoru K., Girardin S.E. (2014). NOD proteins: Regulators of inflammation in health and disease. Nat. Rev. Immunol..

[100] Song D.H., Lee J.-O. (2012). Sensing of microbial molecular patterns by Toll-like receptors. Immunol. Rev..

[101] Fukushima K., Sasaki I., Ogawa H., Naito H., Funayama Y., Matsuno S. (1999). Colonization of microflora in mice: Mucosal defense against luminal bacteria. J. Gastroenterol..

[102] McDole J.R., Wheeler L.W., McDonald K.G., Wang B., Konjufca V., Knoop K.A., Newberry R.D., Miller M.J. (2012). Goblet cells deliver luminal antigen to CD103 + dendritic cells in the small intestine. Nature.

[103] McDonald B.D., Jabri B., Bendelac A. (2018). Diverse developmental pathways of intestinal intraepithelial lymphocytes. Nat. Rev. Immunol..

[104] Olivares-Villagómez D., Van Kaer L. (2018). Intestinal Intraepithelial Lymphocytes: Sentinels of the Mucosal Barrier. Trends Immunol..

[105] Mowat A.M., Agace W.W. (2014). Regional specialization within the intestinal immune system. Nat. Rev. Immunol..

[106] Hume D.A., Perry V.H., Gordon S. (1984). The mononuclear phagocyte system of the mouse defined by immunohistochemical localisation of antigen F4/80: Macrophages associated with epithelia. Anat. Rec..

[107] Lee S.-H., Starkey P.M., Gordon S. (1985). Quantitative analysis of total macrophage content in adult mouse tissues. Imrnunochemical Studies with Monoclonal Antibody F4/80. J. Exp. Med..

[108] Smythies L.E., Sellers M., Clements R.H., Mosteller-Barnum M., Meng G., Benjamin W.H., Orenstein J.M., Smith P.D. (2005). Human intestinal macrophages display profound inflammatory anergy despite avid phagocytic and bacteriocidal activity. J. Clin. Investig..

[109] Kumawat A.K., Yu C., Mann E.A., Schridde A., Finnemann S.C., Mowat A.M. (2018). Expression and characterization of αvβ5 integrin on intestinal macrophages. Eur. J. Immunol..

[110] Schridde A., Bain C.C., Mayer J.U., Montgomery J., Pollet E., Denecke B., Milling S.W.F., Jenkins S.J., Dalod M., Henri S. (2017). Tissue-specific differentiation of colonic macrophages requires TGFβ receptor-mediated signaling. Mucosal Immunol..

[111] Smith P.D., Ochsenbauer-Jambor C., Smythies L.E. (2005). Intestinal macrophages: Unique effector cells of the innate immune system. Immunol. Rev..

[112] Gordon S. (1995). The macrophage. BioEssays.

[113] Müller A.J., Kaiser P., Dittmar K.E.J., Weber T.C., Haueter S., Endt K., Songhet P., Zellweger C., Kremer M., Fehling H.-J. (2012). Salmonella Gut Invasion Involves TTSS-2-Dependent Epithelial Traversal, Basolateral Exit, and Uptake by Epithelium-Sampling Lamina Propria Phagocytes. Cell Host Microbe.

[114] Montalban-Arques A., Chaparro M., Gisbert J.P., Bernardo D. (2018). The Innate Immune System in the Gastrointestinal Tract: Role of Intraepithelial Lymphocytes and Lamina Propria Innate Lymphoid Cells in Intestinal Inflammation. Inflamm. Bowel Dis..

[115] Artis D., Spits H. (2015). The biology of innate lymphoid cells. Nature.

[116] Cella M., Fuchs A., Vermi W., Facchetti F., Otero K., Lennerz J.K.M., Doherty J.M., Mills J.C., Colonna M. (2009). A human natural killer cell subset provides an innate source of IL-22 for mucosal immunity. Nature.

[117] Satoh-Takayama N., Vosshenrich C.A.J., Lesjean-Pottier S., Sawa S., Lochner M., Rattis F., Mention J.-J., Thiam K., Cerf-Bensussan N., Mandelboim O. (2008). Microbial Flora Drives Interleukin 22 Production in Intestinal NKp46+ Cells that Provide Innate Mucosal Immune Defense. Immunity.

[118] Zook E.C., Kee B.L. (2016). Development of innate lymphoid cells. Nat. Immunol..

[119] Klose C.S.N., Artis D. (2016). Innate lymphoid cells as regulators of immunity, inflammation and tissue homeostasis. Nat. Immunol..

[120] Hori S., Nomura T., Sakaguchi S. (2003). Control of Regulatory T Cell Development by the Transcription Factor Foxp3. Science.

[121] Makita S., Kanai T., Oshima S., Uraushihara K., Totsuka T., Sawada T., Nakamura T., Koganei K., Fukushima T., Watanabe M. (2004). CD4+CD25bright T Cells in Human Intestinal Lamina Propria as Regulatory Cells. J. Immunol..

[122] Brandtzaeg P., Johansen F.-E. (2005). Mucosal B cells: Phenotypic characteristics, transcriptional regulation, and homing properties. Immunol. Rev..

[123] Macpherson A.J., McCoy K.D., Johansen F.-E., Brandtzaeg P. (2008). The immune geography of IgA induction and function. Mucosal Immunol..

[124] Reboldi A., Cyster J.G. (2016). Peyer’s patches: Organizing B-cell responses at the intestinal frontier. Immunol. Rev..

[125] Jung H.C., Eckmann L., Yang S.K., Panja A., Fierer J., Morzycka-Wroblewska E., Kagnoff M.F. (1995). A Distinct Array of Proinflammatory Cytokines is Expressed in Human Colon Epithelial Cells in Response to Bacterial Invasion. J. Clin. Investig..

[126] Johansson M.E., Gustafsson J.K., Holmén-Larsson J., Jabbar K.S., Xia L., Xu H., Ghishan F.K., Carvalho F.A., Gewirtz A.T., Sjövall H. (2014). Bacteria penetrate the normally impenetrable inner colon mucus layer in both murine colitis models and patients with ulcerative colitis. Gut.

[127] Capaldo C.T., Nusrat A. (2009). Cytokine regulation of tight junctions. Biochim. Biophys. Acta Biomembr..

[128] McGee D.W., Vitkus S.J.D., Lee P. (1996). The Effect of Cytokine Stimulation on IL-1 Receptor mRNA Expression by Intestinal Epithelial Cells. Cell. Immunol..

[129] Langerholc T., Maragkoudakis P.A., Wollgast J., Gradisnik L., Cencic A. (2011). Novel and established intestinal cell line models—An indispensable tool in food science and nutrition. Trends Food Sci. Technol..

[130] Sambruy Y., Ferruzza S., Ranaldi G., De Angelis I. (2001). Intestinal Cell Culture Models: Applications in Toxicology and Pharmacology. Cell Biol. Toxicol..

[131] Manda G., Mocanu M.A., Marin D.E., Taranu I. (2015). Dual Effects Exerted in vitro by Micromolar Concentrations of Deoxynivalenol on Undifferentiated Caco-2 Cells. Toxins.

[132] Vandenbroucke V., Croubels S., Martel A., Verbrugghe E., Goossens J., Van Deun K., Boyen F., Thompson A., Shearer N., De Backer P. (2011). The Mycotoxin Deoxynivalenol Potentiates Intestinal Inflammation by Salmonella Typhimurium in Porcine Ileal Loops. PLoS ONE.

[133] Lee M., Vasioukhin V. (2008). Cell polarity and cancer—Cell and tissue polarity as a non-canonical tumor suppressor. J. Cell Sci..

[134] Hauck W., Stanners C.P. (1991). Control of Carcinoembryonic Antigen Gene Family Expression in a Differentiating Colon Carcinoma Cell Line, Caco-2. Cancer Res..

[135] Ude V.C., Brown D.M., Viale L., Kanase N., Stone V., Johnston H.J. (2017). Impact of copper oxide nanomaterials on differentiated and undifferentiated Caco-2 intestinal epithelial cells; assessment of cytotoxicity, barrier integrity, cytokine production and nanomaterial penetration. Part. Fibre Toxicol..

[136] Cheng K.-C., Li C., Uss A.S. (2008). Prediction of oral drug absorption in humans – from cultured cell lines and experimental animals. Expert Opin. Drug Metab. Toxicol..

[137] Fogh J., Trempe G., Fogh J. (1975). New Human Tumor Cell Lines. Human Tumor Cells in vitro.

[138] Lea T., Verhoeckx K., Cotter P., López-Expósito I., Kleiveland C., Lea T., Mackie A., Requena T., Swiatecka D., Wichers H. (2015). Caco-2 Cell Line. The Impact of Food Bioactives on Health: In Vitro and Ex Vivo Models.

[139] Pearce S.C., Coia H.G., Karl J.P., Pantoja-Feliciano I.G., Zachos N.C., Racicot K. (2018). Intestinal in vitro and ex vivo Models to Study Host-Microbiome Interactions and Acute Stressors. Front. Physiol..

[140] Ponce de León-Rodríguez M.C., Guyot J.-P., Laurent-Babot C. (2019). Intestinal in vitro cell culture models and their potential to study the effect of food components on intestinal inflammation. Crit. Rev. Food Sci. Nutr..

[141] Hidalgo I.J., Raub T.J., Borchardt R.T. (1989). Characterization of the human colon carcinoma cell line (Caco-2) as a model system for intestinal epithelial permeability. Gastroenterology.

[142] Furrie E., Macfarlane S., Thomson G., Macfarlane G.T. (2005). Toll-like receptors-2, -3 and -4 expression patterns on human colon and their regulation by mucosal-associated bacteria. Immunology.

[143] Diesing A.-K., Nossol C., Panther P., Walk N., Post A., Kluess J., Kreutzmann P., Dänicke S., Rothkötter H.-J., Kahlert S. (2011). Mycotoxin deoxynivalenol (DON) mediates biphasic cellular response in intestinal porcine epithelial cell lines IPEC-1 and IPEC-J2. Toxicol. Lett..

[144] Nossol C., Barta-Böszörményi A., Kahlert S., Zuschratter W., Faber-Zuschratter H., Reinhardt N., Ponsuksili S., Wimmers K., Diesing A.-K., Rothkötter H.-J. (2015). Comparing Two Intestinal Porcine Epithelial Cell Lines (IPECs): Morphological Differentiation, Function and Metabolism. PLoS ONE.

[145] Koh S.Y., George S., Brözel V., Moxley R., Francis D., Kaushik R.S. (2008). Porcine intestinal epithelial cell lines as a new in vitro model for studying adherence and pathogenesis of enterotoxigenic Escherichia coli. Vet. Microbiol..

[146] Bertero A., Spicer L.J., Caloni F. (2018). Fusarium mycotoxins and in vitro species-specific approach with porcine intestinal and brain in vitro barriers: A review. Food Chem. Toxicol..

[147] Campbell J.J., Davidenko N., Caffarel M.M., Cameron R.E., Watson C.J. (2011). A Multifunctional 3D Co-Culture System for Studies of Mammary Tissue Morphogenesis and Stem Cell Biology. PLoS ONE.

[148] Bertero A., Augustyniak J., Buzanska L., Caloni F. (2019). Species-specific models in toxicology: In vitro epithelial barriers. Environ. Toxicol. Pharmacol..

[149] Tremblay E., Auclair J., Delvin E., Levy E., Ménard D., Pshezhetsky A.V., Rivard N., Seidman E.G., Sinnett D., Vachon P.H. (2006). Gene expression profiles of normal proliferating and differentiating human intestinal epithelial cells: A comparison with the Caco-2 cell model. J. Cell. Biochem..

[150] Mahler G.J., Shuler M.L., Glahn R.P. (2009). Characterization of Caco-2 and HT29-MTX cocultures in an in vitro digestion/cell culture model used to predict iron bioavailability. J. Nutr. Biochem..

[151] Smith M.-C., Gheux A., Coton M., Madec S., Hymery N., Coton E. (2018). In vitro co-culture models to evaluate acute cytotoxicity of individual and combined mycotoxin exposures on Caco-2, THP-1 and HepaRG human cell lines. Chem. Biol. Interact..

[152] Akbari P., Braber S., Gremmels H., Koelink P.J., Verheijden K.A.T., Garssen J., Fink-Gremmels J. (2014). Deoxynivalenol: A trigger for intestinal integrity breakdown. FASEB J..

[153] Pierron A., Mimoun S., Murate L.S., Loiseau N., Lippi Y., Bracarense A.-P.F.L., Liaubet L., Schatzmayr G., Berthiller F., Moll W.-D. (2016). Intestinal toxicity of the masked mycotoxin deoxynivalenol-3-β-d-glucoside. Arch. Toxicol..

[154] Bony S., Carcelen M., Olivier L., Devaux A. (2006). Genotoxicity assessment of deoxynivalenol in the Caco-2 cell line model using the Comet assay. Toxicol. Lett..

[155] Kouadio J.H., Mobio T.A., Baudrimont I., Moukha S., Dano S.D., Creppy E.E. (2005). Comparative study of cytotoxicity and oxidative stress induced by deoxynivalenol, zearalenone or fumonisin B1 in human intestinal cell line Caco-2. Toxicology.

[156] Ji J., Wang Q., Wu H., Xia S., Guo H., Blaženović I., Zhang Y., Sun X. (2019). Insights into cellular metabolic pathways of the combined toxicity responses of Caco-2 cells exposed to deoxynivalenol, zearalenone and Aflatoxin B1. Food Chem. Toxicol..

[157] Diesing A.-K., Nossol C., Dänicke S., Walk N., Post A., Kahlert S., Rothkötter H.-J., Kluess J. (2011). Vulnerability of Polarised Intestinal Porcine Epithelial Cells to Mycotoxin Deoxynivalenol Depends on the Route of Application. PLoS ONE.

[158] Goossens J., Pasmans F., Verbrugghe E., Vandenbroucke V., De Baere S., Meyer E., Haesebrouck F., De Backer P., Croubels S. (2012). Porcine intestinal epithelial barrier disruption by the Fusariummycotoxins deoxynivalenol and T-2 toxin promotes transepithelial passage of doxycycline and paromomycin. BMC Vet. Res..

[159] Broekaert N., Devreese M., Demeyere K., Berthiller F., Michlmayr H., Varga E., Adam G., Meyer E., Croubels S. (2016). Comparative in vitro cytotoxicity of modified deoxynivalenol on porcine intestinal epithelial cells. Food Chem. Toxicol..

[160] Liao P., Liao M., Li L., Tan B., Yin Y. (2017). Effect of deoxynivalenol on apoptosis, barrier function, and expression levels of genes involved in nutrient transport, mitochondrial biogenesis and function in IPEC-J2 cells. Toxicol. Res..

[161] Wan L.Y.M., Turner P.C., El-Nezami H. (2013). Individual and combined cytotoxic effects of Fusarium toxins (deoxynivalenol, nivalenol, zearalenone and fumonisins B1) on swine jejunal epithelial cells. Food Chem. Toxicol..

[162] Wang X., Zhang Y., Zhao J., Cao L., Zhu L., Huang Y., Chen X., Rahman S.U., Feng S., Li Y. (2019). Deoxynivalenol Induces Inflammatory Injury in IPEC-J2 Cells via NF-κB Signaling Pathway. Toxins.

[163] Taranu I., Braicu C., Marin D.E., Pistol G.C., Motiu M., Balacescu L., Beridan Neagoe I., Burlacu R. (2015). Exposure to zearalenone mycotoxin alters in vitro porcine intestinal epithelial cells by differential gene expression. Toxicol. Lett..

[164] Marin D.E., Motiu M., Taranu I. (2015). Food Contaminant Zearalenone and Its Metabolites Affect Cytokine Synthesis and Intestinal Epithelial Integrity of Porcine Cells. Toxins.

[165] Zhang J., Zheng N., Liu J., Li F.D., Li S.L., Wang J.Q. (2015). Aflatoxin B1 and aflatoxin M1 induced cytotoxicity and DNA damage in differentiated and undifferentiated Caco-2 cells. Food Chem. Toxicol..

[166] Romero A., Ares I., Ramos E., Castellano V., Martínez M., Martínez-Larrañaga M.-R., Anadón A., Martínez M.-A. (2016). Mycotoxins modify the barrier function of Caco-2 cells through differential gene expression of specific claudin isoforms: Protective effect of illite mineral clay. Toxicology.

[167] Guerra M.C., Galvano F., Bonsi L., Speroni E., Costa S., Renzulli C., Cervellati R. (2005). Cyanidin-3-O-b-glucopyranoside, a natural free-radical scavenger against aflatoxin B1- and ochratoxin A-induced cell damage in a human hepatoma cell line (Hep G2) and a human colonic adenocarcinoma cell line (CaCo-2). Br. J. Nutr..

[168] Bovdisova I., Grabacka M., Capcarova M. (2016). Interaction of citrinin and resveratrol and their effect on Caco-2 cell growth. J. Cent. Eur. Agric..

[169] Salah A., Bouaziz C., Prola A., Silva J.P.D., Bacha H., Abid-Essefi S., Lemaire C. (2017). Citrinin induces apoptosis in human HCT116 colon cancer cells through endoplasmic reticulum stress. J. Toxicol. Environ. Health A.

[170] Assunção R., Pinhão M., Loureiro S., Alvito P., Silva M.J. (2019). A multi-endpoint approach to the combined toxic effects of patulin and ochratoxin a in human intestinal cells. Toxicol. Lett..

[171] Hanelt M., Gareis M., Kollarczik B. (1994). Cytotoxicity of mycotoxins evaluated by the MTT-cell culture assay. Mycopathologia.

[172] Schneeberger K., Roth S., Nieuwenhuis E.E.S., Middendorp S. (2018). Intestinal epithelial cell polarity defects in disease: Lessons from microvillus inclusion disease. Dis. Model. Mech..

[173] Khamchun S., Thongboonkerd V. (2018). Cell cycle shift from G0/G1 to S and G2/M phases is responsible for increased adhesion of calcium oxalate crystals on repairing renal tubular cells at injured site. Cell Death Discov..

[174] Maresca M., Mahfoud R., Pfohl-Leszkowicz A., Fantini J. (2001). The Mycotoxin Ochratoxin A Alters Intestinal Barrier and Absorption Functions but Has No Effect on Chloride Secretion. Toxicol. Appl. Pharmacol..

[175] Yang H., Chung D.H., Kim Y.B., Choi Y.H., Moon Y. (2008). Ribotoxic mycotoxin deoxynivalenol induces G2/M cell cycle arrest via p21Cip/WAF1 mRNA stabilization in human epithelial cells. Toxicology.

[176] Pucci B., Kasten M., Giordano A. (2000). Cell Cycle and Apoptosis. Neoplasia.

[177] Bensassi F., Gallerne C., Sharaf el dein O., Hajlaoui M.R., Lemaire C., Bacha H. (2014). In vitro investigation of toxicological interactions between the fusariotoxins deoxynivalenol and zearalenone. Toxicon.

[178] Odenwald M.A., Turner J.R. (2013). Intestinal Permeability Defects: Is It Time to Treat?. Clin. Gastroenterol. Hepatol..

[179] Madara J.L. (1998). Regulation of the movement of solutes across tight junctions. Annu. Rev. Physiol..

[180] Jimison L.H., Tria S.A., Khodagholy D., Gurfinkel M., Lanzarini E., Hama A., Malliaras G.G., Owens R.M. (2012). Measurement of Barrier Tissue Integrity with an Organic Electrochemical Transistor. Adv. Mater..

[181] González-Mariscal L., Domínguez-Calderón A., Raya-Sandino A., Ortega-Olvera J.M., Vargas-Sierra O., Martínez-Revollar G. (2014). Tight junctions and the regulation of gene expression. Semin. Cell Dev. Biol..

[182] Maresca M., Mahfoud R., Garmy N., Fantini J. (2002). The Mycotoxin Deoxynivalenol Affects Nutrient Absorption in Human Intestinal Epithelial Cells. J. Nutr..

[183] Van De Walle J., Sergent T., Piront N., Toussaint O., Schneider Y.-J., Larondelle Y. (2010). Deoxynivalenol affects in vitro intestinal epithelial cell barrier integrity through inhibition of protein synthesis. Toxicol. Appl. Pharmacol..

[184] Pinton P., Nougayrède J.-P., Del Rio J.-C., Moreno C., Marin D.E., Ferrier L., Bracarense A.-P., Kolf-Clauw M., Oswald I.P. (2009). The food contaminant deoxynivalenol, decreases intestinal barrier permeability and reduces claudin expression. Toxicol. Appl. Pharmacol..

[185] McLaughlin J., Padfield P.J., Burt J.P.H., O’Neill C.A. (2004). Ochratoxin A increases permeability through tight junctions by removal of specific claudin isoforms. Am. J. Physiol. Cell Physiol..

[186] Ranaldi G., Mancini E., Ferruzza S., Sambuy Y., Perozzi G. (2007). Effects of red wine on ochratoxin A toxicity in intestinal Caco-2/TC7 cells. Toxicol. Vitr..

[187] Watson P.M.D., Paterson J.C., Thom G., Ginman U., Lundquist S., Webster C.I. (2013). Modelling the endothelial blood-CNS barriers: A method for the production of robust in vitromodels of the rat blood-brain barrier and blood-spinal cord barrier. BMC Neurosci..

[188] Srinivasan B., Kolli A.R., Esch M.B., Abaci H.E., Shuler M.L., Hickman J.J. (2015). TEER Measurement Techniques for In vitro Barrier Model Systems. J. Lab. Autom..

[189] Ling K.-H., Wan M.L.Y., El-Nezami H., Wang M. (2016). Protective Capacity of Resveratrol, a Natural Polyphenolic Compound, against Deoxynivalenol-Induced Intestinal Barrier Dysfunction and Bacterial Translocation. Chem. Res. Toxicol..

[190] Schwanhäusser B., Busse D., Li N., Dittmar G., Schuchhardt J., Wolf J., Chen W., Selbach M. (2011). Global quantification of mammalian gene expression control. Nature.

[191] Vogel C., De Sousa Abreu R., Ko D., Le S.-Y., Shapiro B.A., Burns S.C., Sandhu D., Boutz D.R., Marcotte E.M., Penalva L.O. (2010). Sequence signatures and mRNA concentration can explain two-thirds of protein abundance variation in a human cell line. Mol. Syst. Biol..

[192] Lambert D., Padfield P.J., McLaughlin J., Cannell S., O’Neill C.A. (2007). Ochratoxin A displaces claudins from detergent resistant membrane microdomains. Biochem. Biophys. Res. Commun..

[193] Pinton P., Braicu C., Nougayrede J.-P., Laffitte J., Taranu I., Oswald I.P. (2010). Deoxynivalenol Impairs Porcine Intestinal Barrier Function and Decreases the Protein Expression of Claudin-4 through a Mitogen-Activated Protein Kinase-Dependent Mechanism. J. Nutr..

[194] Maresca M., Yahi N., Younès-Sakr L., Boyron M., Caporiccio B., Fantini J. (2008). Both direct and indirect effects account for the pro-inflammatory activity of enteropathogenic mycotoxins on the human intestinal epithelium: Stimulation of interleukin-8 secretion, potentiation of interleukin-1β effect and increase in the transepithelial passage of commensal bacteria. Toxicol. Appl. Pharmacol..

[195] DeMeo M.T., Mutlu E.A., Keshavarzian A., Tobin M.C. (2002). Intestinal permeation and gastrointestinal disease. J. Clin. Gastroenterol..

[196] Pastorelli L., De Salvo C., Mercado J.R., Vecchi M., Pizarro T.T. (2013). Central Role of the Gut Epithelial Barrier in the Pathogenesis of Chronic Intestinal Inflammation: Lessons Learned from Animal Models and Human Genetics. Front. Immunol..

[197] Zhou H.-R., Islam Z., Pestka J.J. (2005). Induction of Competing Apoptotic and Survival Signaling Pathways in the Macrophage by the Ribotoxic Trichothecene Deoxynivalenol. Toxicol. Sci..

[198] Pestka J.J., Uzarski R.L., Islam Z. (2005). Induction of apoptosis and cytokine production in the Jurkat human T cells by deoxynivalenol: Role of mitogen-activated protein kinases and comparison to other 8-ketotrichothecenes. Toxicology.

[199] Oh S.-Y., Boermans H.J., Swamy H.V.L.N., Sharma B.S., Karrow N.A. (2012). Immunotoxicity of Penicillium Mycotoxins on Viability and Proliferation of Bovine Macrophage Cell Line (BOMACs). Open Mycol. J..

[200] Wan L.-Y.M., Woo C.-S.J., Turner P.C., Wan J.M.-F., El-Nezami H. (2013). Individual and combined effects of Fusarium toxins on the mRNA expression of pro-inflammatory cytokines in swine jejunal epithelial cells. Toxicol. Lett..

[201] Van De Walle J., Romier B., Larondelle Y., Schneider Y.-J. (2008). Influence of deoxynivalenol on NF-κB activation and IL-8 secretion in human intestinal Caco-2 cells. Toxicol. Lett..

[202] Vandenbroucke V., Croubels S., Verbrugghe E., Boyen F., Backer P.D., Ducatelle R., Rychlik I., Haesebrouck F., Pasmans F. (2009). The mycotoxin deoxynivalenol promotes uptake of Salmonella Typhimurium in porcine macrophages, associated with ERK1/2 induced cytoskeleton reorganization. Vet. Res..

[203] Oh S.-Y., Mead P.J., Sharma B.S., Quinton V.M., Boermans H.J., Smith T.K., Swamy H.V.L.N., Karrow N.A. (2015). Effect of Penicillium mycotoxins on the cytokine gene expression, reactive oxygen species production, and phagocytosis of bovine macrophage (BoMacs) function. Toxicol. Vitr..

[204] Devreese M., Backer P.D., Croubels S. (2013). Different methods to counteract mycotoxin production and its impact on animal health. Vlaams Diergeneeskd. Tijdschr..

[205] Faucet-Marquis V., Joannis-Cassan C., Hadjeba-Medjdoub K., Ballet N., Pfohl-Leszkowicz A. (2014). Development of an in vitro method for the prediction of mycotoxin binding on yeast-based products: Case of aflatoxin B1, zearalenone and ochratoxin A. Appl. Microbiol. Biotechnol..

[206] Kabak B., Dobson A.D.W., Var I. (2006). Strategies to Prevent Mycotoxin Contamination of Food and Animal Feed: A Review. Crit. Rev. Food Sci. Nutr..

[207] Peng W.-X., Marchal J.L.M., Van der Poel A.F.B. (2018). Strategies to prevent and reduce mycotoxins for compound feed manufacturing. Anim. Feed Sci. Technol..

[208] Horky P., Skalickova S., Baholet D., Skladanka J. (2018). Nanoparticles as a Solution for Eliminating the Risk of Mycotoxins. Nanomaterials.

[209] Abdel-Wahhab M.A., Nada S.A., Khalil F.A. (2002). Physiological and toxicological responses in rats fed aflatoxin-contaminated diet with or without sorbent materials. Anim. Feed Sci. Technol..

[210] Avantaggiato G., Havenaar R., Visconti A. (2003). Assessing the zearalenone-binding activity of adsorbent materials during passage through a dynamic in vitro gastrointestinal model. Food Chem. Toxicol..

[211] Huwig A., Freimund S., Käppeli O., Dutler H. (2001). Mycotoxin detoxication of animal feed by different adsorbents. Toxicol. Lett..

[212] Ramos A.-J., Fink-Gremmels J., Hernández E. (1996). Prevention of Toxic Effects of Mycotoxins by Means of Nonnutritive Adsorbent Compounds. J. Food Prot..

[213] Boudergue C., Burel C., Dragacci S., Favrot M.-C., Fremy J.-M., Massimi C., Prigent P., Debongnie P., Pussemier L., Boudra H. (2009). Review of mycotoxin-detoxifying agents used as feed additives: Mode of action, efficacy and feed/food safety. EFSA Supporting Publ..

[214] Avantaggiato G., Solfrizzo M., Visconti A. (2005). Recent advances on the use of adsorbent materials for detoxification of Fusarium mycotoxins. Food Addit. Contam..

[215] Phillips T.D., Afriyie-Gyawu E., Williams J., Huebner H., Ankrah N.-A., Ofori-Adjei D., Jolly P., Johnson N., Taylor J., Marroquin-Cardona A. (2008). Reducing human exposure to aflatoxin through the use of clay: A review. Food Addit. Contam. Part A.

[216] Karlovsky P. (2011). Biological detoxification of the mycotoxin deoxynivalenol and its use in genetically engineered crops and feed additives. Appl. Microbiol. Biotechnol..

[217] Döll S., Dänicke S., Valenta H., Flachowsky G. (2004). In vitro studies on the evaluation of mycotoxin detoxifying agents for their efficacy on deoxynivalenol and zearalenone. Arch. Anim. Nutr..

[218] Kolosova A., Stroka J. (2012). Evaluation of the effect of mycotoxin binders in animal feed on the analytical performance of standardised methods for the determination of mycotoxins in feed. Food Addit. Contam. Part A.

[219] Jouany J.P. (2007). Methods for preventing, decontaminating and minimizing the toxicity of mycotoxins in feeds. Anim. Feed Sci. Technol..

[220] Kogan G., Kocher A. (2007). Role of yeast cell wall polysaccharides in pig nutrition and health protection. Livest. Sci..

[221] Pereyra C.M., Cavaglieri L.R., Chiacchiera S.M., Dalcero A. (2013). The corn influence on the adsorption levels of aflatoxin B1 and zearalenone by yeast cell wall. J. Appl. Microbiol..

[222] Shetty P.H., Jespersen L. (2006). Saccharomyces cerevisiae and lactic acid bacteria as potential mycotoxin decontaminating agents. Trends Food Sci. Technol..

[223] Yiannikouris A., André G., Buléon A., Jeminet G., Canet I., François J., Bertin G., Jouany J.-P. (2004). Comprehensive Conformational Study of Key Interactions Involved in Zearalenone Complexation with β-d-Glucans. Biomacromolecules.

[224] Yiannikouris A., André G., Poughon L., François J., Dussap C.-G., Jeminet G., Bertin G., Jouany J.-P. (2006). Chemical and Conformational Study of the Interactions Involved in Mycotoxin Complexation with β-d-Glucans. Biomacromolecules.

[225] Binder E.M. (2007). Managing the risk of mycotoxins in modern feed production. Anim. Feed Sci. Technol..

[226] Firmin S., Gandia P., Morgavi D.P., Houin G., Jouany J.P., Bertin G., Boudra H. (2010). Modification of aflatoxin B1 and ochratoxin A toxicokinetics in rats administered a yeast cell wall preparation. Food Addit. Contam. Part A.

[227] Firmin S., Morgavi D.P., Yiannikouris A., Boudra H. (2011). Effectiveness of modified yeast cell wall extracts to reduce aflatoxin B1 absorption in dairy ewes. J. Dairy Sci..

[228] Yiannikouris A., Kettunen H., Apajalahti J., Pennala E., Moran C.A. (2013). Comparison of the sequestering properties of yeast cell wall extract and hydrated sodium calcium aluminosilicate in three in vitro models accounting for the animal physiological bioavailability of zearalenone. Food Addit. Contam. Part A.

[229] Shetty P.H., Hald B., Jespersen L. (2007). Surface binding of aflatoxin B1 by Saccharomyces cerevisiae strains with potential decontaminating abilities in indigenous fermented foods. Int. J. Food Microbiol..

[230] Ringot D., Lerzy B., Chaplain K., Bonhoure J.-P., Auclair E., Larondelle Y. (2007). In vitro biosorption of ochratoxin A on the yeast industry by-products: Comparison of isotherm models. Bioresour. Technol..

[231] Sabater-Vilar M., Malekinejad H., Selman M.H.J., Van der Doelen M.A.M., Fink-Gremmels J. (2007). In vitro assessment of adsorbents aiming to prevent deoxynivalenol and zearalenone mycotoxicoses. Mycopathologia.

[232] Joannis-Cassan C., Tozlovanu M., Hadjeba-Medjdoub K., Ballet N., Pfohl-Leszkowicz A. (2011). Binding of zearalenone, aflatoxin B1, and ochratoxin A by yeast-based products: A method for quantification of adsorption performance. J. Food Prot..

[233] Gerbaldo G.A., Barberis C., Pascual L., Dalcero A., Barberis L. (2012). Antifungal activity of two Lactobacillus strains with potential probiotic properties. FEMS Microbiol. Lett..

[234] Hahn I., Kunz-Vekiru E., Twarużek M., Grajewski J., Krska R., Berthiller F. (2015). Aerobic and anaerobic in vitro testing of feed additives claiming to detoxify deoxynivalenol and zearalenone. Food Addit. Contam. Part A.

[235] Cavret S., Laurent N., Videmann B., Mazallon M., Lecoeur S. (2010). Assessment of deoxynivalenol (DON) adsorbents and characterisation of their efficacy using complementary in vitro tests. Food Addit. Contam. Part A.

[236] Oh S.-Y., Quinton V.M., Boermans H.J., Swamy H.V.L.N., Karrow N.A. (2015). In vitro exposure of Penicillium mycotoxins with or without a modified yeast cell wall extract (mYCW) on bovine macrophages (BoMacs). Mycotoxin Res..

[237] Avantaggiato G., Havenaar R., Visconti A. (2004). Evaluation of the intestinal absorption of deoxynivalenol and nivalenol by an in vitro gastrointestinal model, and the binding efficacy of activated carbon and other adsorbent materials. Food Chem. Toxicol..

[238] Prapapanpong J., Udomkusonsri P., Mahavorasirikul W., Choochuay S., Tansakul N. (2019). In vitro studies on gastrointestinal monogastric and avian models to evaluate the binding efficacy of mycotoxin adsorbents by liquid chromatography-tandem mass spectrometry. J. Adv. Vet. Anim. Res..

[239] Natsch A., Gfeller H., Emter R., Ellis G. (2010). Use of in vitro testing to identify an unexpected skin sensitizing impurity in a commercial product: A case study. Toxicol. Vitr. Int. J. Publ. Assoc. BIBRA.

[240] Trapecar M., Cencic A. (2012). Application of Gut Cell Models for Toxicological and Bioactivity Studies of Functional and Novel Foods. Foods.

[241] Zeissig S., Bürgel N., Günzel D., Richter J., Mankertz J., Wahnschaffe U., Kroesen A.J., Zeitz M., Fromm M., Schulzke J.-D. (2007). Changes in expression and distribution of claudin 2, 5 and 8 lead to discontinuous tight junctions and barrier dysfunction in active Crohn’s disease. Gut.

[242] Lemke S.L., Grant P.G., Phillips T.D. (1998). Adsorption of Zearalenone by Organophilic Montmorillonite Clay. J. Agric. Food Chem..

[243] Pan F., Han L., Zhang Y., Yu Y., Liu J. (2015). Optimization of Caco-2 and HT29 co-culture in vitro cell models for permeability studies. Int. J. Food Sci. Nutr..

[244] Borenfreund E., Puerner J.A. (1985). Toxicity determined in vitro by morphological alterations and neutral red absorption. Toxicol. Lett..

[245] Cencič A., Langerholc T. (2010). Functional cell models of the gut and their applications in food microbiology—A review. Int. J. Food Microbiol..

[246] Suzuki K., Oida T., Hamada H., Hitotsumatsu O., Watanabe M., Hibi T., Yamamoto H., Kubota E., Kaminogawa S., Ishikawa H. (2000). Gut Cryptopatches: Direct Evidence of Extrathymic Anatomical Sites for Intestinal T Lymphopoiesis. Immunity.

[247] Trapecar M., Leouffre T., Faure M., Jensen H.E., Granum P.E., Cencic A., Hardy S.P. (2011). The use of a porcine intestinal cell model system for evaluating the food safety risk of Bacillus cereus probiotics and the implications for assessing enterotoxigenicity. APMIS.

[248] Vamadevan A.S., Fukata M., Arnold E.T., Thomas L.S., Hsu D., Abreu M.T. (2010). Regulation of Toll-like receptor 4-associated MD-2 in intestinal epithelial cells: A comprehensive analysis. Innate Immun..

[249] Tavelin S., Taipalensuu J., Soderberg L., Morrison R., Chong S., Artursson P. (2003). Prediction of the Oral Absorption of Low-Permeability Drugs Using Small Intestine-Like 2/4/A1 Cell Monolayers. Pharm. Res..

[250] Nakayama H., Kitagawa N., Otani T., Iida H., Anan H., Inai T. (2018). Ochratoxin A, citrinin and deoxynivalenol decrease claudin-2 expression in mouse rectum CMT93-II cells. Microscopy.

[251] Artursson P., Palm K., Luthman K. (2001). Caco-2 monolayers in experimental and theoretical predictions of drug transport1PII of original article: S0169-409X(96)00415-2. The article was originally published in Advanced Drug Delivery Reviews 22 (1996) 67–84.1. Adv. Drug Deliv. Rev..

[252] Klarić M.Š. (2012). Adverse Effects Of Combined Mycotoxins / Štetni Učinci Kombiniranih Mikotoksina. Arch. Ind. Hyg. Toxicol..

[253] Kleiveland C.R., Verhoeckx K., Cotter P., López-Expósito I., Kleiveland C., Lea T., Mackie A., Requena T., Swiatecka D., Wichers H. (2015). Co-culture Caco-2/Immune Cells. The Impact of Food Bioactives on Health: In Vitro and Ex Vivo Models.

[254] Fitzgerald K.A., Malhotra M., Curtin C.M., O’ Brien F.J., O’ Driscoll C.M. (2015). Life in 3D is never flat: 3D models to optimise drug delivery. J. Control. Release.

[255] Goers L., Freemont P., Polizzi K.M. (2014). Co-culture systems and technologies: Taking synthetic biology to the next level. J. R. Soc. Interface.

[256] Thomsen L.B., Burkhart A., Moos T. (2015). A Triple Culture Model of the Blood-Brain Barrier Using Porcine Brain Endothelial cells, Astrocytes and Pericytes. PLoS ONE.

[257] Cheli F., Campagnoli A., Dell’Orto V. (2013). Fungal populations and mycotoxins in silages: From occurrence to analysis. Anim. Feed Sci. Technol..

[258] Richard E., Heutte N., Bouchart V., Garon D. (2009). Evaluation of fungal contamination and mycotoxin production in maize silage. Anim. Feed Sci. Technol..

[259] Rodrigues I. (2014). A review on the effects of mycotoxins in dairy ruminants. Anim. Prod. Sci..

[260] Malmuthuge N., Griebel P.J., Guan L.L. (2015). The Gut Microbiome and Its Potential Role in the Development and Function of Newborn Calf Gastrointestinal Tract. Front. Vet. Sci..

[261] Kruber P., Trump S., Behrens J., Lehmann I. (2011). T-2 toxin is a cytochrome P450 1A1 inducer and leads to MAPK/p38- but not aryl hydrocarbon receptor-dependent interleukin-8 secretion in the human intestinal epithelial cell line Caco-2. Toxicology.

